# Activation of EphrinB2/EphB2 signaling in the spine cord alters glia-neuron interactions in mice with visceral hyperalgesia following maternal separation

**DOI:** 10.3389/fphar.2024.1463339

**Published:** 2024-09-03

**Authors:** Shufen Guo, Yu Wang, Qingling Duan, Wei Gu, Qun Fu, Zhengliang Ma, Jiaping Ruan

**Affiliations:** ^1^ Department of Anesthesiology, Nanjing Drum Tower Hospital Clinical College of Nanjing University of Chinese Medicine, Nanjing, Jiangsu, China; ^2^ Department of Anesthesiology, Nanjing Drum Tower Hospital, Affiliated Hospital of Medical School, Nanjing University, Nanjing, Jiangsu, China

**Keywords:** visceral hyperalgesia, maternal separation, ephrinB2/ephB2, glia-neuron, NMDA receptor

## Abstract

**Background:**

Sress early in life has been linked to visceral hyperalgesia and associated functional gastrointestinal disorders. In a mouse model of visceral hyperalgesia, we investigated whether the EphB2 receptor and its EphrinB2 ligand in spinal cord contribute to dysregulation of glia-neuron interactions.

**Methods:**

An established mouse model of stress due to maternal separation (MS). Pups were separated from their mothers for 14 days during early development, then analyzed several weeks later in terms of visceral sensitivity based on the abdominal withdrawal reflex score and in terms of expression of c-fos, EphrinB2, EphB2, and phosphorylated MAP kinases (ERK, p38, JNK).

**Results:**

Visceral hyperalgesia due to MS upregulated EphB2, EphrinB2 and c-fos in the spinal cord, and c-fos levels positively correlated with those of EphB2 and EphrinB2. Spinal astrocytes, microglia, and neurons showed upregulation of EphB2, EphrinB2 and phosphorylated MAP kinases. Blocking EphrinB2/EphB2 signaling in MS mice reduced visceral sensitivity, activation of neurons and glia, and phosphorylation of NMDA receptor. Activating EphrinB2/EphB2 signaling in unstressed mice induced visceral hyperalgesia, upregulation of c-fos, and activation of NMDA receptor similar to maternal separation.

**Conclusion:**

The stress of MS during early development may lead to visceral hyperalgesia by upregulating EphrinB2/EphB2 in the spinal cord and thereby altering neuron-glia interactions.

## Introduction

Irritable bowel syndrome and other functional gastrointestinal disorders usually involve visceral hyperalgesia, in which nerves in the gut are hypersensitive to stimuli (Chey, Kurlander, and Eswaran, 2015). A strong risk factor for visceral hyperalgesia is stress early in life (Hu et al., 2020), but what molecules and pathways mediate the relationship between the two are poorly understood. What is known is that the dorsal root ganglion sends visceral pain signals to the dorsal horn in the spine (Louwies et al., 2019), which transmits the signals further to neural networks involved in nociception (Moloney et al., 2015). Also known is that two types of glia, microglia and astrocytes, can be activated by stress (Ji, Berta, and Nedergaard, 2013) to release pro-inflammatory cytokines that regulate neuronal activity (Milligan and Watkins, 2009) in a way that triggers and maintains visceral hyperalgesia (Dodds et al., 2016; Song et al., 2014). Unknown is how stress alters glia-neuron interactions to give rise to chronic hypersensitivity in the gut.

The EphB2 receptors and EphrinB2 ligands are found in peripheral tissues and mature brain, where they play a pivotal role in regulating diverse physiological and pathophysiological processes (Wu et al., 2021; Duan et al., 2024). Peripheral EphrinB2/EphB2 receptor was involved in visceral hypersensitivity in postinfectious irritable bowel syndrome (IBS) rats (Zhang et al., 2019). EphrinB2/EphB2 receptor tyrosine kinases also plays a key role in the structural and functional plasticity of synapses during early neurodevelopment as well as in the mature brain and spinal cord (Lim, Matsuda, and Poo, 2008; Klein and Kania, 2014). EphB2 demonstrates the capacity to orchestrate functional presynaptic specializations while also governing postsynaptic maturation through the promotion of spine morphogenesis and recruitment of neurotransmitter receptors (Kayser et al., 2006; Planagumà et al., 2016). Moreover, EphrinB2/EphB2 mediates heightened neural activation and excitatory synaptic transmission in cases of neuropathic pain (Song et al., 2008). Both EphB2 and EphrinB2 proteins are expressed in neurons and glia, where they help regulate the correct formation of neuronal circuits (Lauterbach and Klein, 2006). Activation of EphrinB2/EphB2 signaling in the spine can lead to hyperalgesia by altering neural development and synaptic plasticity (Bundesen et al., 2003; Chen et al., 2022), apparently through multiple mechanisms dependent on, and independent of, the *N*-methyl-d-aspartate (NMDA) receptor (Ma et al., 2020). Conversely, inhibition of EphB2/EphrinB2 signaling dampens chronic pain in animal models of inflammatory, neuropathic, and cancer pain (Zhou et al., 2015b; Liu et al., 2013). Therefore, we hypothesized that altered signaling involving the EphB2 receptor and its EphrinB2 ligand may lead to altered interactions between glia and neurons in visceral hyperalgesia.

We investigated here the effects of EphrinB2/EphB2 on glia-neuron interactions in the spine in mice that were separated for a defined period from their mothers during early development. In this animal model, the stress arising from maternal separation is known to induce visceral hyperalgesia (Du et al., 2019), which can be measured as abdominal withdrawal reflex after colorectal distension (Li et al., 2023). Among the potential molecules affected by EphrinB2/EphB2 signaling, we focused on c-fos because its upregulation correlates with activation of neurons during nociception (Coggeshall, 2005) and on MAP kinases, because activation of these kinases correlates with activation of EphB2 (Poliakov et al., 2008a) and because we showed previously that MAP kinases mediate at least some of the effects of EphrinB/EphB signaling on synaptic plasticity (Ruan et al., 2010) and nociception (Cao et al., 2008).

## Methods

### Mouse model of visceral hyperalgesia

All animal procedures were approved by the Animal Ethics Committee at Nanjing Medical University. Adult male and female C57BL/6 mice (Laboratory Animal Center of Nanjing Medical University, Nanjing, China) were housed with *ad libitum* access to food and water. Newborn male pups were separated from their dams in the same cage by an opaque isolation board for 3 h daily from 9:00 to 12:00 h from postnatal days 2–15, then the board was removed and pups were returned to their dams (Du et al., 2019). Control pups were raised under the same conditions except that no isolation board was introduced into the cage. All pups were weaned and separated from their dams on postnatal day 21, and analyzed as described below starting at 8 weeks of age.

The numbers of animals and replicate measurements in different experiments were based on our previous experience (Ruan et al., 2010; Cao et al., 2008).

### Effects of inhibiting or activating EphrinB2/EphB2 signaling in the mouse model

Mice that experienced maternal separation or not were anesthetized intraperitoneally using sodium pentobarbital (40 mg/kg), then injected intrathecally with one of the following: a chimera of EphB2 with Fc (catalog no. 467-B2, R&D Systems, Minnesota, United States), which binds to EphrinB2 and prevents it from stimulating endogenous EphB2; a chimera of EphrinB2 and Fc (catalog no. 496-EB, R&D Systems, Minnesota, United States), which binds and activates endogenous EphB2; or, as a negative control, recombinant Fc from human IgG1 (catalog no. 110-HG, R&D Systems, Minnesota, United States). Injections EphB2-Fc or EphrinB2-Fc(0.1-µg or 0.5-µg/5 μL, i. t., daily for three consecutive days) were made between vertebrae L5 and L6 using a 25-µL Hamilton microinjector (Ruan et al., 2010), and tail flicking was interpreted as successful puncture. Injections were delivered within 30 s, the needle was left in place for 15 s, then the needle was gently removed. All doses of the drug are based on the results of the pre-experimental studies in reference to Zhou et al. (2015a). The detailed dose and treatment time point for each drug are given in the illustration section.

### Assessment of visceral sensitivity

Visceral sensitivity was quantified by scoring the abdominal withdrawal reflex following colorectal distention (Li et al., 2023). Mice were lightly anesthetized with 1%–1.5% isoflurane, then a rubber balloon was carefully inserted into the colorectum 3 cm from the anus and secured to the base of the tail using hypoallergenic silk tape. Animals were placed individually into a clear box (20 × 10 × 18 cm), allowed to habituate for 10–15 min, then the balloon was inflated sequentially for 20 s at pressures of 20, 40 and 60 mmHg. The balloon was deflated for 2–3 min between inflations. The abdominal withdrawal reflex was scored from 0 to 4 as described (Chen et al., 2022) by investigators blinded to mouse grouping. Scores were averaged from triplicate measurements on each animal.

In immunohistochemistry, immunofluorescence staining and Western blotting experiments to compare activation or expression of key proteins between mice that experienced maternal separation or not (see below), colorectal distension stimulation was performed at 60 mmHg every 3 min during 15 min. We chose these conditions to avoid damaging the colorectal tissue, given that the balloon had to be inflated at 60 mmHg in order to induce nociceptive behaviors.

### Immunohistochemistry against key proteins in the spine

Mice were anesthetized under isoflurane before transcardial perfusion and injected with saline and 4% paraformaldehyde, then the spinal cord was removed and sliced into sections 20 µm thick using a cryostat (CM1800, Leica, Germany). Sections were washed in phosphate-buffered saline (PBS), blocked in goat serum for 30 min, then incubated with primary antibodies against the following proteins: EphB2 (1:200; catalog no. MABN726, Merck & Millipore, United States), EphrinB2 (1:100; catalog no. HPA008999, Merck & Millipore, United States), phospho-ERK (1:200; catalog no. sc-136521, Santa Cruz, Dallas, United States), phospho-p38 (1:200; catalog no. sc-166182, Santa Cruz, Dallas, United States), phospho-JNK (1:200; catalog no. sc-6254, Santa Cruz, Dallas, United States), c-fos (1:500; catalog no. sc-166940, Santa Cruz, Dallas, United States), GFAP (1:200; catalog no. sc-33673, Santa Cruz, Dallas, United States), NeuN (1:1500; catalog no. ABN90, Merck & Millipore, United States), and Iba1 (1:200; catalog no. sc-32725, Santa Cruz, Dallas, United States). Subsequently, the sections were rinsed three times with PBS and incubated for 2 h with anti-rabbit Alexa Fluor 488 (1:500, Invitrogen, California, United States) or anti-mouse Alexa Fluor 555 (1:500, Invitrogen, California, United States) antibodies. After washing with PBS for 3 times, incubate with DAPI.

Immunostained sections were examined under Olympus microscope (1–71, Olympus, Japan) and images were analyzed using ImageJ software (National Institutes of Health, United States).

### Western blotting against key proteins in the spine

Under isoflurane anesthesia, mice were sacrificed, the spinal cord was removed, flash-frozen in liquid nitrogen, then thawed and lysed in RIPA lysis buffer. Equal amounts of total protein (30 µg) were fractionated by SDS-PAGE and transferred onto polyvinylidene difluoride membranes, which were immersed in blocking buffer (Beyotime, Shanghai, China), then incubated with primary antibodies against the following proteins: EphB2 (1:500; catalog no. MABN726, Merck & Millipore, United States), EphrinB2 (1:500; catalog no. HPA008999, Merck & Millipore, United States), phospho-ERK (1:500; catalog no. sc-136521, Santa Cruz, Dallas, United States), ERK (1:500; catalog no. sc-514302, Santa Cruz, Dallas, United States), phospho-p38 (1:500; catalog no. sc-166182, Santa Cruz, Dallas, United States), p38 (1:500; catalog no. sc-7972, Santa Cruz, Dallas, United States), phospho-JNK (1:500; catalog no. sc-6254, Santa Cruz, Dallas, United States), JNK (1:500; catalog no. sc-137019, Santa Cruz, Dallas, United States), GFAP (1:200; catalog no. sc-33673, Santa Cruz, Dallas, United States), NeuN (1:2000; catalog no. ABN90, Merck & Millipore, United States), Iba1 (1:500; catalog no. sc-32725, Santa Cruz, Dallas, United States), NR2B (1:1000; catalog no. 42125, Cell Signaling Technology, Massachusetts, United States), phospho-NR2B (TYR1472) (1:1000; catalog no.4208, Cell Signaling Technology, Massachusetts, United States), c-fos (1:1000; catalog no. sc-166940, Santa Cruz, Dallas, United States), and Actin (1:2000; catalog no. sc-8432, Santa Cruz, Dallas, United States). Membranes were washed three times with Tris-buffered saline containing 0.1% Tween-20 (TBST), then incubated for 2 h at room temperature with horseradish peroxidase-conjugated goat secondary antibodies against mouse IgG (1:5000; catalog no. AP124P, Merck & Millipore, United States) or rabbit IgG (1:5000; catalog no. AP132P, Merck & Millipore, United States). Membranes were again washed three times with TBST, images were scanned with Micro Chemi (DNR Bio-imaging systems, Jerusalem, Israel) and the optical densities of detected bands were quantified using Image software J (US National Institutes of Health, Bethesda, MD, United States).

### Statistical analysis

Data were analyzed and figures prepared using Graph Pad Prism 9.0.0 (Inc., San Diego, CA). Data were reported as mean ± SEM unless stated otherwise. Pairwise differences were assessed for significance using the two-tailed unpaired Student’s t-test, while differences among three or more groups were assessed using one- or two-way analysis of variance. Differences associated with *p* < 0.05 were considered significant.

## Results

### Visceral hyperalgesia due to maternal separation involves upregulation of EphB2, EphrinB2 and c-fos in the spine

**FIGURE 1:**
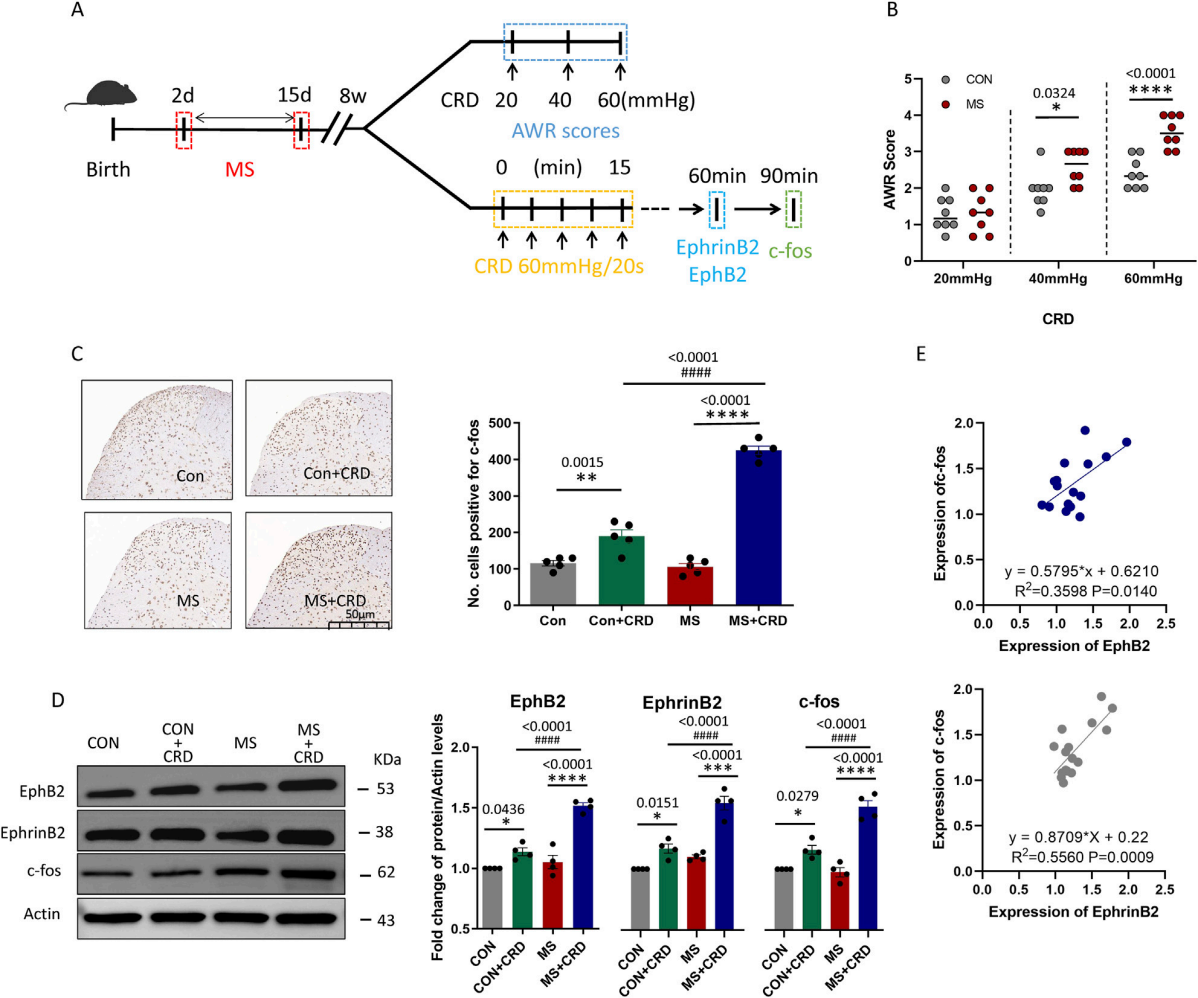
Maternal separation early in development causes visceral hyperalgesia in response to colorectal distension. **(A)** Schematic illustration of the experimental protocol. CRD, colorectal distension; MS, maternal separation **(B)** Score on abdominal withdrawal reflex after colorectal distension. Quantification is shown for eight animals per condition. CON, control. *P < 0.05, ****P < 0.0001 vs. CON, based on two-way repeated-measures ANOVA, followed by the Bonferroni multiple-comparisons test. **(C)** Representative thin sections of spinal cord after immunostaining against c-fos and quantification of cells expressing c-fos. CRD induced Fos protein expression in superficial lamine of spinal cord, we count the c-fos^+^ neurons in lamine I-V of spinal cord of view at magnification ×10 and average the results. Results are shown for five animals per condition. Scale bar, 50 µm. Based on one-way ANOVA and the Bonferroni multiple-comparisons test: **P < 0.01, ****P < 0.0001 between CON and CON + CRD or between MS and MS + CRD; ^####^ *P* < 0.0001 between CON + CRD and MS + CRD. **(D)** Representative Western blotting of total lysate of spine tissue and quantification of EphB2 and EphrinB2. Protein levels were normalized to those of Actin in the same sample, and the relative protein level in the control group without CRD was defined as 1. Quantification is shown for four animals per treatment. Based on one-way ANOVA and the Bonferroni multiple-comparisons test: *P < 0.05, ****P < 0.0001 between CON and CON + CRD or between MS and MS + CRD; ^####^ *P* < 0.0001 between CON + CRD and MS + CRD. **(E)** Correlation between levels of c-fos and levels of EphB2 or EphrinB2 based on Western blotting of total lysates of spine tissue.

Mice were subjected to colorectal distension (CRD) following maternal separation (MS). The schematic diagram and time course of CRD combined with abdominal withdrawal reflex (AWR) score under pressures of 20, 40, and 60 mmHg are shown in Figure 1A **.** The records confirmed that separating mouse pups from their mothers for a 14-day period soon after birth led to visceral hyperalgesia several weeks later, which we detected as an increase in abdominal withdrawal reflex in response to colorectal distension, the AWR score of MS mice did not change at 20 mmHg but increased significantly at 40 and 60 mmHg (Figure 1B). The stronger reflex was associated with upregulation of c-fos (Figure 1C), which signals neuronal activation during nociception (Coggeshall, 2005), as well as with upregulation of EphB2 and EphrinB2 (Figure 1D). The time points for the detection of EphrinB2/EphB2, and Fos protein expression are set at 60 min and 90 min after CRD, respectively. In fact, levels of c-fos correlated positively with those of EphB2 and EphrinB2 (Figure 1E). These results suggest that stress due to maternal separation induces visceral hyperalgesia by activating EphrinB2/EphB2 signaling.

### Visceral hyperalgesia involves activation of MAP kinases in spine cells in which EphB2 is upregulated

**FIGURE 2:**
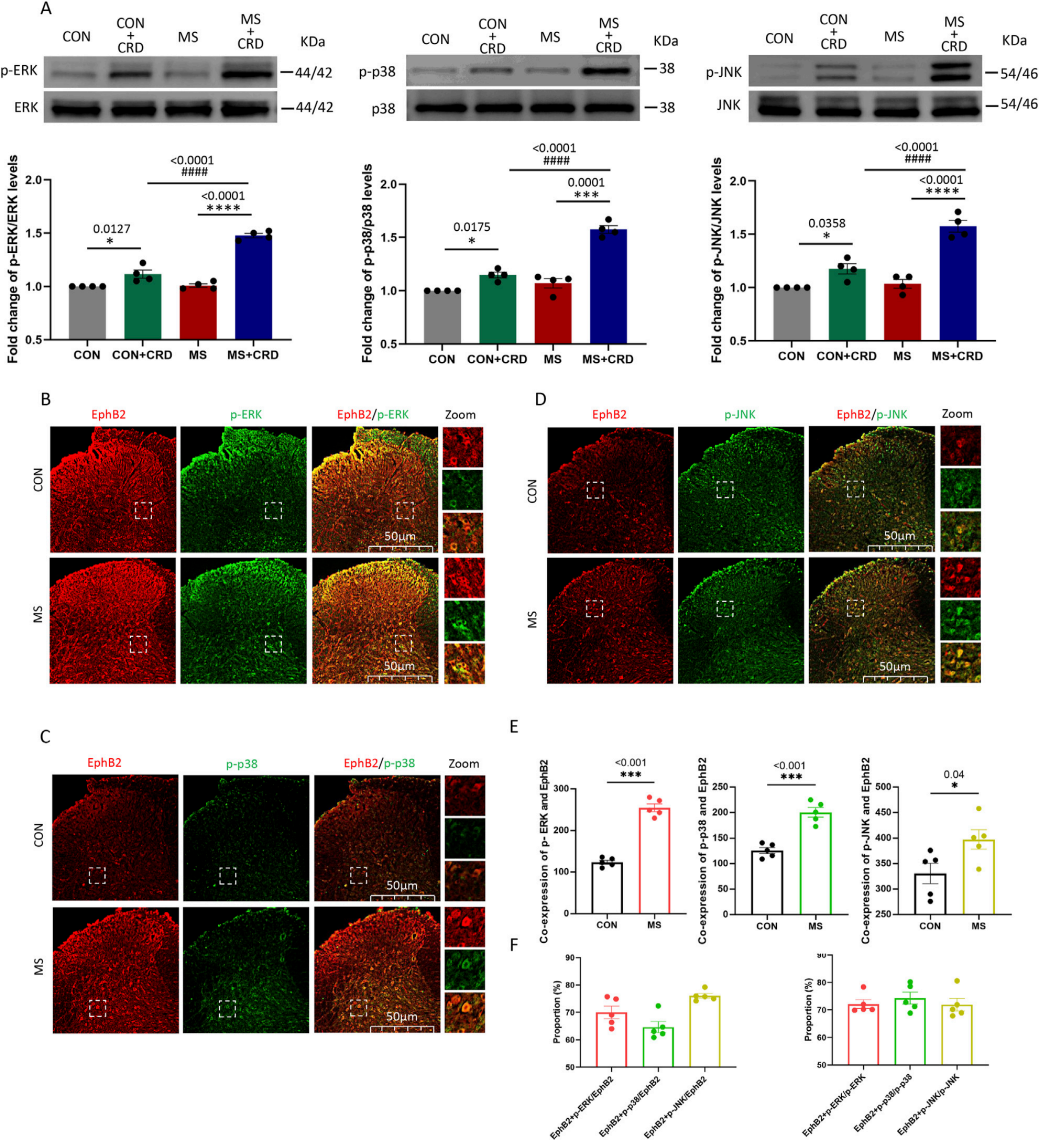
Visceral hyperalgesia in response to colorectal distension activates EphrinB2/EphB2 signaling and downstream MAP kinases. Mice were subjected to maternal separation (MS) early in development or not (CON), then later subjected to colorectal distension (CRD) or not. **(A)** Representative western blots of total lysate from spinal cord and quantification of the active, phosphorylated forms of the MAP kinases ERK, p38 and JNK. Levels of phosphorylated protein were normalized to those of the corresponding total protein, and the relative level of phosphorylated protein in the control group without CRD was defined as 1. Quantification is shown for four animals per condition. Based on one-way ANOVA and the Bonferroni multiple-comparisons test: *P < 0.05, ****P < 0.0001 between CON and CON + CRD or between MS and MS + CRD; ^####^ *P* < 0.0001 between CON + CRD and MS + CRD. **(B–D)** Immunostaining of thin sections of spinal cord against EphB2 (red) and the phosphorylated forms of p38, ERK, or JNK (green). Scale bar, 50 µm. The boxed regions in the large images are shown at higher magnification on the *far right* (“Zoom”). **(E)** Co-expression of spinal EphB2 and MAPKs. Based on one-way ANOVA and the Bonferroni multiple-comparisons test: *P < 0.05, ***P < 0.001 between CON and MS. **(F)** Proportion of spinal cord cells expressing each activated MAP kinase that also expressed EphB2 (*left plot*) or proportion of spinal cord cells expressing EphB2 that also expressed each of the phosphorylated MAP kinases (*right plot*).

Visceral hyperalgesia due to maternal separation was associated with activation of three MAP kinases known to act downstream of EphB2: ERK, p38 and JNK (Figure 2A). Immunofluorescence staining of thin sections from the spine showed that EphB2-positive cells in the spinal cord mainly co-expressed with these kinases 60min after CRD (Figures 2B–D). In the control group, we observed a small amount of EphB2 or MAPKs activation, but the co-expression of these receptors were significant less than those in MS group (Figure 2E). The ratio of EphB2 + p-ERK, EphB2 + p-p38 and EphB2 + p-JNK to EphB2 was 70.02%, 61.46% and 76.05%, respectively (Figure 2F, left). While the ratio of EphB2 + p-ERK to p-ERK, EphB2 + p-p38 to p-p38 and EphB2 + p-JNK to p-JNK was 72.16%,74.36% and 71.93%, respectively (Figure 2F, right). These results suggest that the stress caused by maternal separation stimulates EphrinB2/EphB2 signaling, which in turn activates MAP kinases.

### Visceral hyperalgesia involves activation of neurons, astrocytes and microglia in the dorsal horn of the spine

**FIGURE 3:**
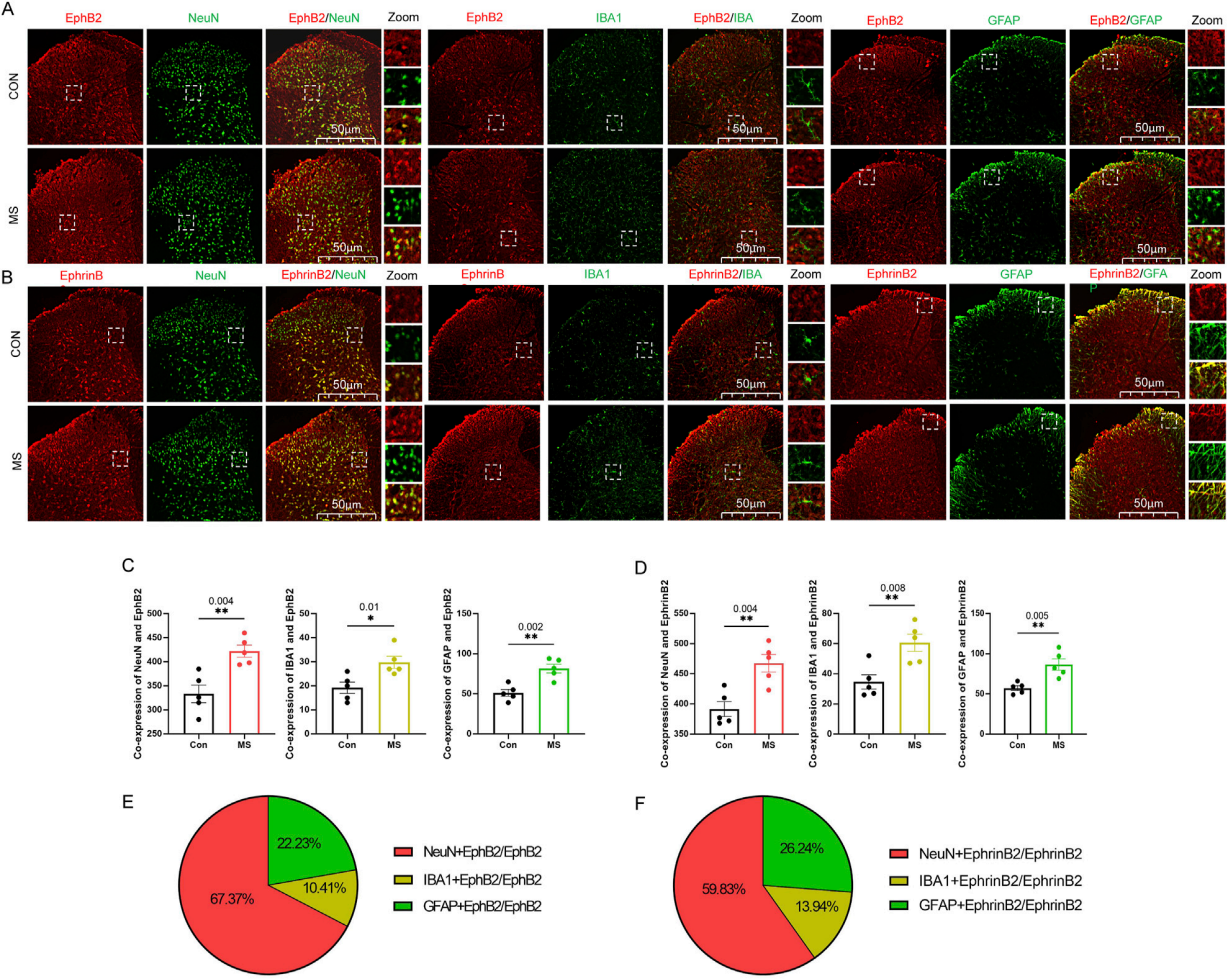
Expression of EphrinB2 and EphB2 in neurons, astrocytes and microglia in spinal cord. **(A, B)** Mice that with (MS) or without (Con) experienced maternal separation were subjected to colorectal distension, then the spinal cord was thin-sectioned and immunostained against EphB2 **(A)** or EphrinB2 **(B)** and a marker for neurons (NeuN), activated microglia (Iba1), or astrocytes (GFAP). Scale bar, 50 µm. The boxed regions in the large images are shown at higher magnification on the *far right* (“Zoom”). **(C, D)** Co-expression of spinal EphB2 or EphrinB2 and NeuN, Iba1or GFAP. Based on one-way ANOVA and the Bonferroni multiple-comparisons test: *P < 0.05, **P < 0.01 between CON and MS. **(E, F)** The proportion of EphB2 **(E)** or EphrinB2**(F)** activation in neurons (NeuN), microglia (Iba1), or astrocytes (GFAP).

Visceral hyperalgesia due to maternal separation was associated with upregulation of EphB2 and EphrinB2 in three key cell types in the dorsal horn of the spine: neurons, which we identified based on immunostaining against NeuN; astrocytes, based on immunostaining against GFAP; and activated microglia, based on immunostaining against Iba1 in the dorsal horn at 60min (Figures 3A, B). We also observed that a small amount of EphB2 or EphrinB2 was activated in neurons, microglia or astrocytes in the control group, but the amount of co-expression was significantly lower than those in the MS group (Figures 3C, D). The proportion of EphB2 activation in neurons, microglia and astrocytes was 67.37%, 10.14% and 22.23%, respectively. While the proportion of EphrinB2 activation in neurons, microglia and astrocytes was 59.83%, 13.94% and 26.24%, respectively (Figures 3E, F). These results suggest that visceral hyperalgesia induced by MS activates the EphrinB2/EphB2 signaling pathway in neurons and is accompanied by activation of this signaling pathway in microglia and astrocytes.

### Inhibition of EphrinB2/EphB2 signaling mitigates the effects of maternal separation

**FIGURE 4:**
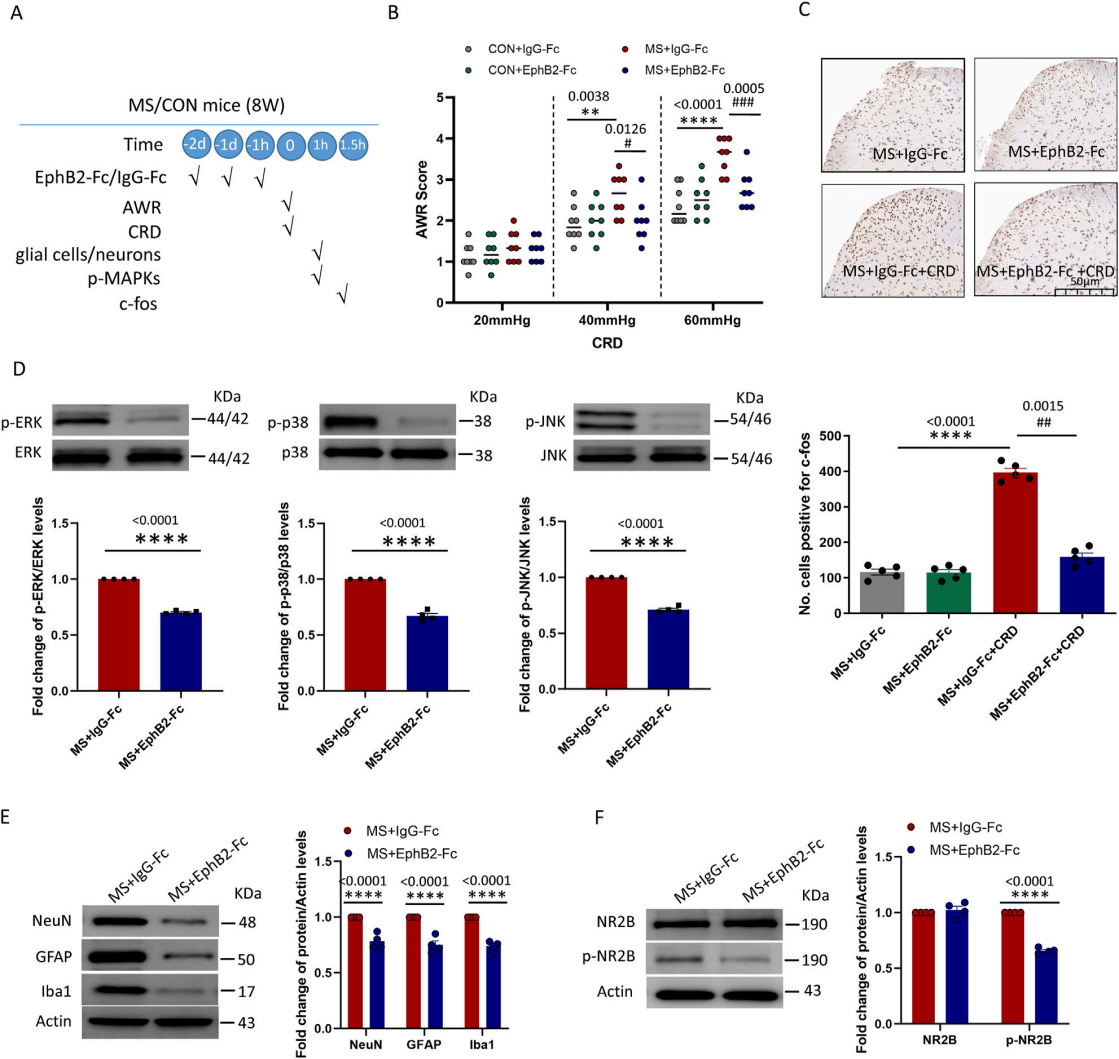
Inhibition of EphrinB2/EphB2 signaling mitigates the effects of maternal separation. **(A)** Schematic illustration of the experimental protocol. Mice were subjected to maternal separation (MS) early in development or not (CON), intrathecally injected with a chimera of EphB2 and Fc (EphB2-Fc) that inhibits EphB2 signaling or a negative-control chimera (IgG-Fc), then subjected to colorectal distension (CRD), during which the abdominal withdrawal reflex (AWR) was measured. Subsequently, the spinal cord was analyzed by Western blotting and immunohistochemistry to observe expression of key proteins. **(B)** AWR score during CRD. Quantification is shown for eight animals per condition. Based on two-way repeated-measure ANOVA and the Bonferroni multiple-comparisons test: **P < 0.01, ****P < 0.0001 between CON + IgG-Fc and MS + IgG-Fc; ^#^ *P* < 0.05, ^###^ *P* < 0.001 between MS + IgG-Fc and MS + EphB2-Fc. **(C)** Representative micrographs of spinal cord tissue after immunostaining against c-fos and quantification of the number of cells expressing c-fos. We count the c-fos + neurons in lamine I-V of spinal cord of view at magnification ×10 and average the results. Results are shown for five animals per condition. Scale bar, 50 µm. Based on one-way ANOVA and the Bonferroni multiple-comparisons test: ****P < 0.0001 between MS + IgG-Fc and MS + IgG-Fc + CRD; ^##^ *P* < 0.01 between MS + IgG-Fc + CRD and MS + EphB2-Fc + CRD. **(D–F)** Representative western blots of total lysate from spinal cord and quantification of **(D)** the active, phosphorylated forms of the MAP kinases ERK, p38 and JNK; **(E)** the cell type markers NeuN, GFAP and Iba1; or **(F)** NR2B and NR2B phosphorylated on Tyr1472. Levels of phosphorylated protein were normalized to those of the corresponding total protein, while levels of markers, NR2B or phospho-NR2B were normalized to those of Actin; the resulting relative protein levels in the MS+IgG-Fc group were defined as 1. Quantification is shown for four animals per condition. Based on unpaired t-test, ****P < 0.0001 compared with MS + IgG-Fc group.

All our experiments so far pointed to EphrinB2/EphB2 signaling as a driver of visceral hyperalgesia due to maternal separation. Consistent with this idea, pretreatment with repeated administration of EphB2-Fc (0.5-µg, i. t., daily for three consecutive days, see experimental protocol in Figure 4A) before CRD inhibited such signaling in mice that had experienced maternal separation mitigated their abdominal withdrawal reflex in response to colorectal distension (Figure 4B), while also downregulating c-fos (Figure 4C). In support of the reliability of our results, inhibiting EphrinB2/EphB2 signaling did not significantly alter the abdominal withdrawal reflex in mice that had not experienced maternal separation, nor did it alter c-fos expression in the absence of colorectal distension.

These therapeutic effects of inhibiting EphrinB2/EphB2 signaling were associated with reduced activation of the three MAP kinases ERK, p38 and JNK in the spine (Figure 4D), as well as lower overall numbers of neurons, astrocytes, and activated microglia (Figure 4E). Given that the NMDA receptor appears to mediate at least some nociceptive signaling by EphrinB/EphB (Liu et al., 2013), we analyzed phosphorylation of the NMDA receptor subunit NR2B in the presence or absence of EphrinB2/EphB2 inhibition. Such inhibition partially reversed the phosphorylation of NR2B induced by colorectal distension, without affecting total levels of NR2B (Figure 4F).

These results support the idea that EphrinB2/EphB2 signaling helps mediate visceral hyperalgesia due to maternal separation, at least in part by stimulating the NMDA receptor.

### Activation of EphrinB2/EphB2 signaling reproduces the effects of maternal separation in naïve mice

**FIGURE 5:**
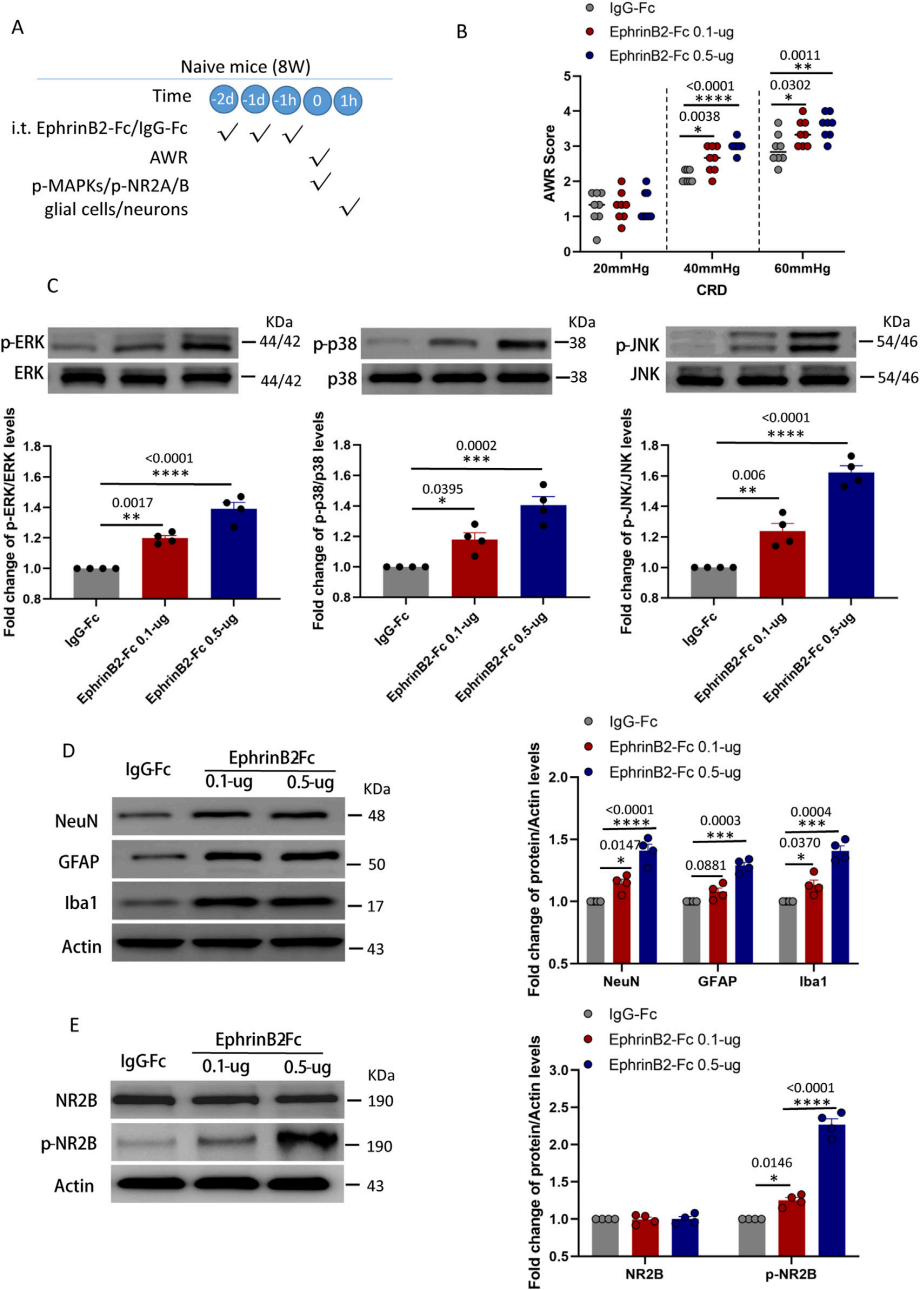
Activation of EphrinB2/EphB2 signaling reproduces the effects of maternal separation in naïve mice. **(A)** Schematic illustration of the experimental protocol. Mice that had not experienced maternal separation early in development were intrathecally injected with a chimera of EphrinB2 and Fc (EphB2-Fc) that activates EphB2 signaling or a negative-control chimera (IgG-Fc), then subjected to colorectal distension (CRD), during which the abdominal withdrawal reflex (AWR) was measured. Subsequently, the spinal cord was analyzed by Western blotting and immunohistochemistry to observe expression of key proteins. **(B)** AWR score during CRD. Quantification is shown for eight animals per condition. Based on two-way repeated-measure ANOVA and the Bonferroni multiple-comparisons test: *P < 0.05, **P < 0.01, ****P < 0.0001 vs IgG-Fc group. **(C)** Representative western blots of total lysate from spinal cord and quantification of the active, phosphorylated forms of the MAP kinases ERK, p38 and JNK. Levels of phosphorylated protein were normalized to those of the corresponding total protein, and the relative level of phosphorylated protein in the IgG-Fc group was defined as 1. Quantification is shown for four animals per condition. Based on one-way ANOVA and Bonferroni multiple comparisons test, *P < 0.05, **P < 0.01, ***P < 0.001, ****P < 0.0001 vs. IgG-Fc group, n = 4 mice in each group. **(D, E)** Representative western blots of total lysate from spinal cord and quantification of **(D)** the cell type markers NeuN, GFAP and Iba1; or **(E)** NR2B and NR2B phosphorylated on Tyr1472. Protein levels were normalized to those of Actin, and the relative protein level in the IgG-Fc group was defined as 1. Quantification is shown for four animals per condition. Based on one-way ANOVA and Bonferroni multiple comparisons test, *P < 0.05, ***P < 0.001, ****P < 0.0001 vs. IgG-Fc group.

The above results suggested that the activation of endogenous EphB2 receptor promotes activation of glial cells and neurons, and produced viscera hyperalgesia after CRD in MS mice. NMDA receptor may contributed to the activation of glial cells and neurons in this process. A corollary of this idea is that activation of EphrinB2/EphB2 signaling should induce changes in naïve mice similar to those observed in animals that experienced maternal separation. We tested this idea by giving naïve mice exogenous EphrinB2 (0.1-µg or 0.5-µg, i. t., daily for three consecutive days, see experimental protocol in Figure 5A). As predicted, the resulting activation of EphrinB2/EphB2 signaling led to visceral hyperalgesia as measured following colorectal distension (Figure 5B), and in the spine it activated the three MAP kinases (Figure 5C); increased numbers of neurons, astrocytes and activated microglia (Figure 5D); and increased levels of phosphorylated NR2B and c-fos (Figure 5E), especially in the higher dose (EphrinB2-Fc 0.5-µg) group. These effects were stronger in animals given the higher dose of exogenous EphrinB2, indicating dose dependence.

These results strongly support a model of visceral hyperalgesia in which EphrinB2/EphB2 signaling in the spine acts *via* MAP kinases to alter interactions among neurons, microglia and astrocytes.

## Discussion

Our work using an established mouse model of early life stress provides evidence of a pathway through which such stress can lead to visceral hyperalgesia later in life. Our experiments suggest a model in which hyperalgesia occurs when activation of EphrinB2/EphB2 signaling alters interactions between glia and neurons in the dorsal horn of the spine through mechanisms at least partly dependent on the NMDA receptor and downstream MAP kinases. Our findings highlight EphrinB2/EphB2 signaling and, more generally, glia-neuron interactions in the dorsal horn as potential therapeutic targets against visceral hyperalgesia arising from stress early in life.

EphrinB2/EphB2 signaling plays important roles in early development of the nervous system (Vasileiou et al., 2013), synaptic plasticity among neurons in the dorsal hone (Liu et al., 2013) and responses to injury (Steinle et al., 2002). At the same time, the signaling pathway contributes to various types of chronic pain (Zhou et al., 2015b; Liu et al., 2013) and, as we show here, to visceral hyperalgesia similar to that in functional gastrointestinal disorders.

We found that the activation of EphrinB2/EphB2 signaling associated with visceral hyperalgesia occurred in astrocytes, microglia cells and neurons in the spine, consistent with the fact that glia and neurons contribute to “central sensitization” in hyperalgesia (Ji et al., 2003; Sengupta, 2009). Indeed, spinal astrocytes and microglia interact with neurons *via* synapses (Song et al., 2014), so their interactions are likely key determinants of central sensitization. For example, activated glia release inflammatory mediators that induce excessive excitatory neuronal activity involving the NMDA receptor (Milligan and Watkins, 2009; Ernst et al., 2019). Activated neurons, for their part, release neurotransmitters that can stimulate glia (Tsuda et al., 2005). Our work justifies deeper analysis of these interactions and how they malfunction in functional gastrointestinal disorders.

We focused on the MAP kinases p38, JNK and ERK because all three are important second messengers in spinal cord during nociception (Zhuang et al., 2006; Poliakov et al., 2008a). *In vitro* studies revealed a dependent positive feedback loop between EphB2 receptors and the Ras-MAPK pathway, that is, EphB2 receptors activate the Ras-MAPK pathway, and the latter increases the amount of EphB2 activation through the dependent positive feedback loop (Liu et al., 2013). The activation of EphB2 is accompanied by the activation of ERK in DRG neurons after nerve injury, and intrathecal administration of EphB2 receptor blocker can inhibit nerve injury-induced pain and ERK activation (Ma et al., 2020; Poliakov et al., 2008b). In this study, we found that EphB2 receptor was highly co-expressed with the three MAPKs. It suggest that the stress caused by maternal separation stimulates EphrinB2/EphB2 signaling, which in turn activates MAP kinases. Moreover, EphrinBs binding to EphBs also induces direct extracellular interaction between EphB and NMDARs (Dalva et al., 2000), resulting in NMDAR aggregation and enhancement of NMDAR dependent Ca^2+^ flux (Takasu et al., 2002). EphB2 controls the function of NMDA receptors (Ma et al., 2020; Shao et al., 2023). Our results add visceral hyperalgesia to the contexts in which EphrinB2/EphB2 signaling has been shown to stimulate NMDA receptor activity.

**FIGURE 6:**
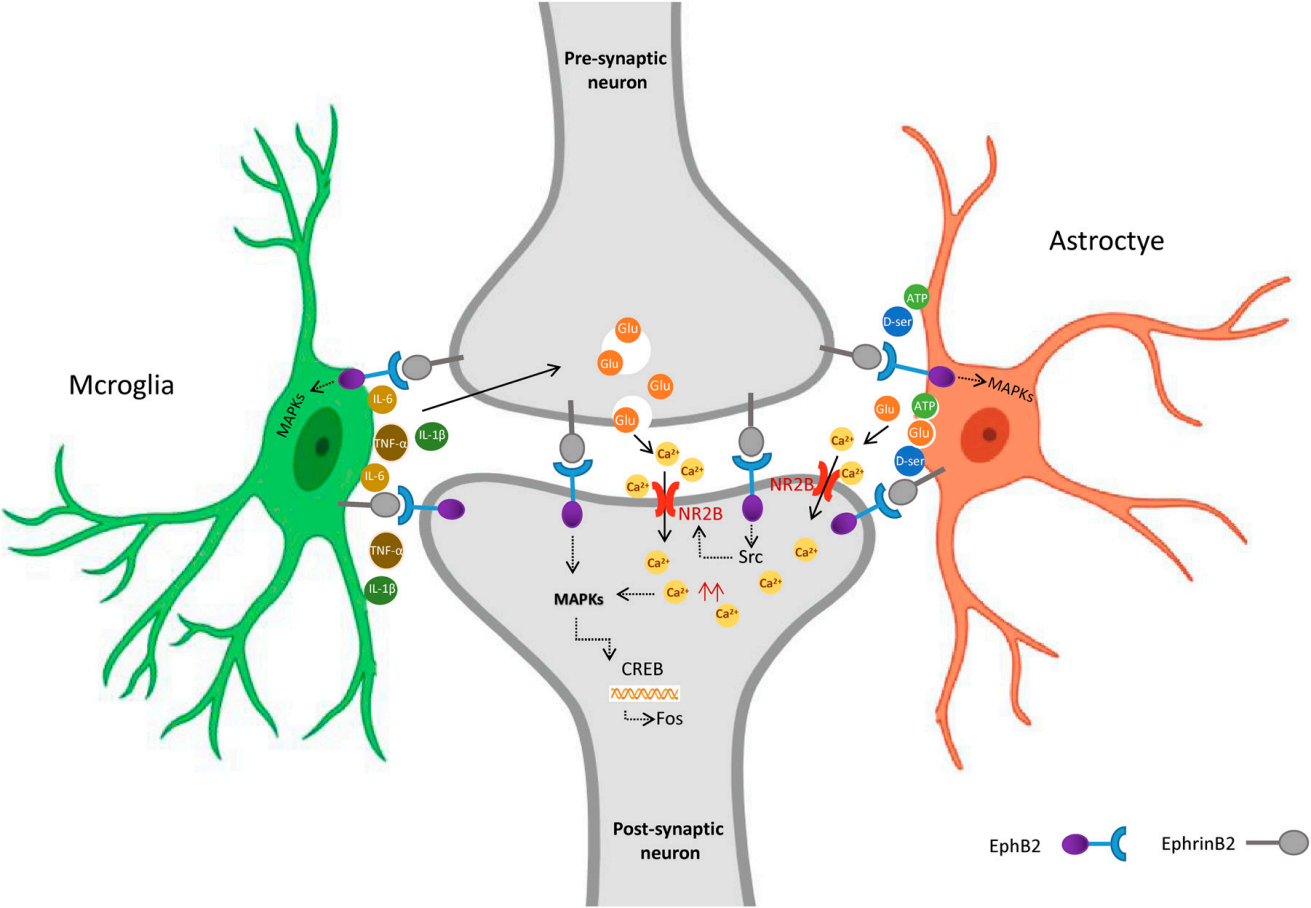
Proposed model for the role of EphrinB2/EphB2 signaling in visceral hyperalgesia due to stress in early life. Activation of spinal EphrinB2/EphB2 signaling alters glia-neuron interactions, which contributes to visceral hyperalgesia arising from the stress of earlier maternal separation. Upon activation of EphrinB2/EphB2 signaling, spinal astrocytes and microglia interact with neurons via synapses, activated glia release inflammatory mediators that induce excessive excitatory neuronal activity involving the NMDA receptor. Figure Created with BioRender.com.

Combined with our and others findings, we propose that in mice exposed to maternal separation, colorectal distension upregulates EphrinB2 in pre-synaptic neurons of the spine, which activates EphB2 in post-synaptic neurons (Figure 6). This activation turns on downstream MAP kinases in the neurons, altering gene expression, perhaps *via* the transcription factors CREB and c-fos. The altered gene expression may affect synapse formation and plasticity (Zhang et al., 2019). In parallel, activated EphB2 on post-synaptic neurons, perhaps acting *via* Src, may induce the NMDA receptor to import Ca^2+^, which stimulates MAP kinases. Given that astrocytes and microglia also express EphrinB2 and EphB2, they may form a “quadruple synapse” in which all 3 cell types interact. Upon activation of EphrinB2/EphB2 signaling, microglia may secrete inflammatory mediators that induce pre-synaptic neurons to release glutamate, which activates NMDA receptors in post-synaptic neurons. Astrocytes, for their part, may secrete gliotransmitters such as glutamate, ATP, and d-serine, which stimulate postsynaptic neuron excitation. Whether MAP kinases mediate some of these effects in microglia and astrocytes remains to be seen, but it seems possible in light of our finding that most MAP kinase activation occurred in spine cells in which EphB2 was upregulated.

The present study has several limitations. Firstly, only the model of visceral hyperalgesia in male mice established by mother separation was investigated, and the potential impact of gender differences on visceral pain within this model was not explored. Secondly, only the effects of blocking or activating EphrinB2/EphB2 on CRD-induced visceral hyperalgesia were assessed, and the potential effects of activating this signaling pathway on the intestinal nervous system and intestinal morphology were not examined. Thirdly, the effect of blocking microglia or astrocytes on neurons has not been explored and should be considered in future studies. Finally,the bidirectional signals of EphrinBs/EphBs were found to be involved in the pro-inflammatory reaction of endothelial cells and leukocytes (Liu et al., 2014). While the activation of EphrinB2 in spinal cord astrocytes played an important role in the pathological changes of amyotrophic lateral sclerosis (ALS) (Urban et al., 2024). Our study only investigated the role of the forward signal activated by the EphB2 receptor, not the role of the reverse signal caused by the activation of EphrinB2. Future studies should further investigate whether the activation of EphrinB2 reverse signaling is involved in visceral hyperalgesia and its mechanism.

## Conclusion

Activation of EphrinB2/EphB2 signaling alters glia-neuron interactions in the spine, which contributes to visceral hyperalgesia arising from the stress of earlier maternal separation. Targeting EphrinB2/EphB2 signaling may be a promising approach for treating stress-induced visceral hyperalgesia in functional gastrointestinal disorders.

## Data Availability

The raw data supporting the conclusions of this article will be made available by the authors, without undue reservation.
