# Corrigendum: Activation of EphrinB2/EphB2 signaling in the spine cord alters glia-neuron interactions in mice with visceral hyperalgesia following maternal separation

**DOI:** 10.3389/fphar.2024.1491784

**Published:** 2024-09-27

**Authors:** Shufen Guo, Yu Wang, Qingling Duan, Wei Gu, Qun Fu, Zhengliang Ma, Jiaping Ruan

**Affiliations:** ^1^ Department of Anesthesiology, Nanjing Drum Tower Hospital Clinical College of Nanjing University of Chinese Medicine, Nanjing, Jiangsu, China; ^2^ Department of Anesthesiology, Nanjing Drum Tower Hospital, Affiliated Hospital of Medical School, Nanjing University, Nanjing, Jiangsu, China

**Keywords:** visceral hyperalgesia, maternal separation, ephrinB2/ephB2, glia-neuron, NMDA receptor

In the published article, there was an error in Figure 1D as published. In Figure 1D “Actin” was incorrectly written as “GAPDH”**.** The corrected Figure 1 and its caption appear below.

**FIGURE 1:**
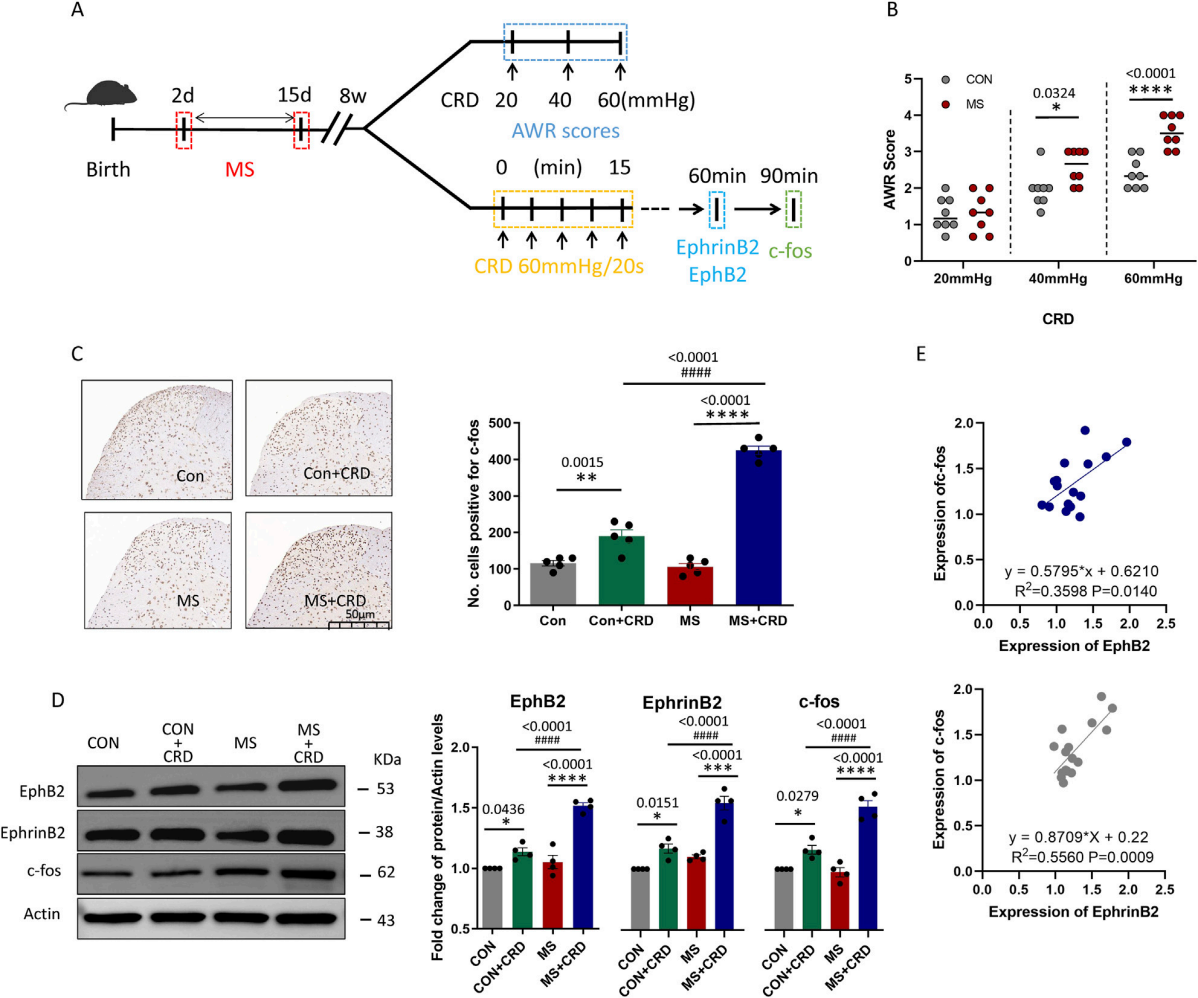
Maternal separation early in development causes visceral hyperalgesia in response to colorectal distension. **(A)** Schematic illustration of the experimental protocol. CRD, colorectal distension; MS, maternal separation **(B)** Score on abdominal withdrawal reflex after colorectal distension. Quantification is shown for eight animals per condition. CON, control. *P < 0.05, ****P < 0.0001 vs. CON, based on two-way repeated-measures ANOVA, followed by the Bonferroni multiple-comparisons test. **(C)** Representative thin sections of spinal cord after immunostaining against c-fos and quantification of cells expressing c-fos. CRD induced Fos protein expression in superficial lamine of spinal cord, we count the c-fos^+^ neurons in lamine I-V of spinal cord of view at magnification ×10 and average the results. Results are shown for five animals per condition. Scale bar, 50 µm. Based on one-way ANOVA and the Bonferroni multiple-comparisons test: **P < 0.01, ****P < 0.0001 between CON and CON + CRD or between MS and MS + CRD; ^####^ *P* < 0.0001 between CON + CRD and MS + CRD. **(D)** Representative Western blotting of total lysate of spine tissue and quantification of EphB2 and EphrinB2. Protein levels were normalized to those of Actin in the same sample, and the relative protein level in the control group without CRD was defined as 1. Quantification is shown for four animals per treatment. Based on one-way ANOVA and the Bonferroni multiple-comparisons test: *P < 0.05, ****P < 0.0001 between CON and CON + CRD or between MS and MS + CRD; ^####^ *P* < 0.0001 between CON + CRD and MS + CRD. **(E)** Correlation between levels of c-fos and levels of EphB2 or EphrinB2 based on Western blotting of total lysates of spine tissue.

In the published article, there was an error in Figure 2E as published. The ordinate “Co-expression of p-JNK and EphB2” in Figure 2E is missing**.** The corrected Figure 2 and its caption appear below.

**FIGURE 2:**
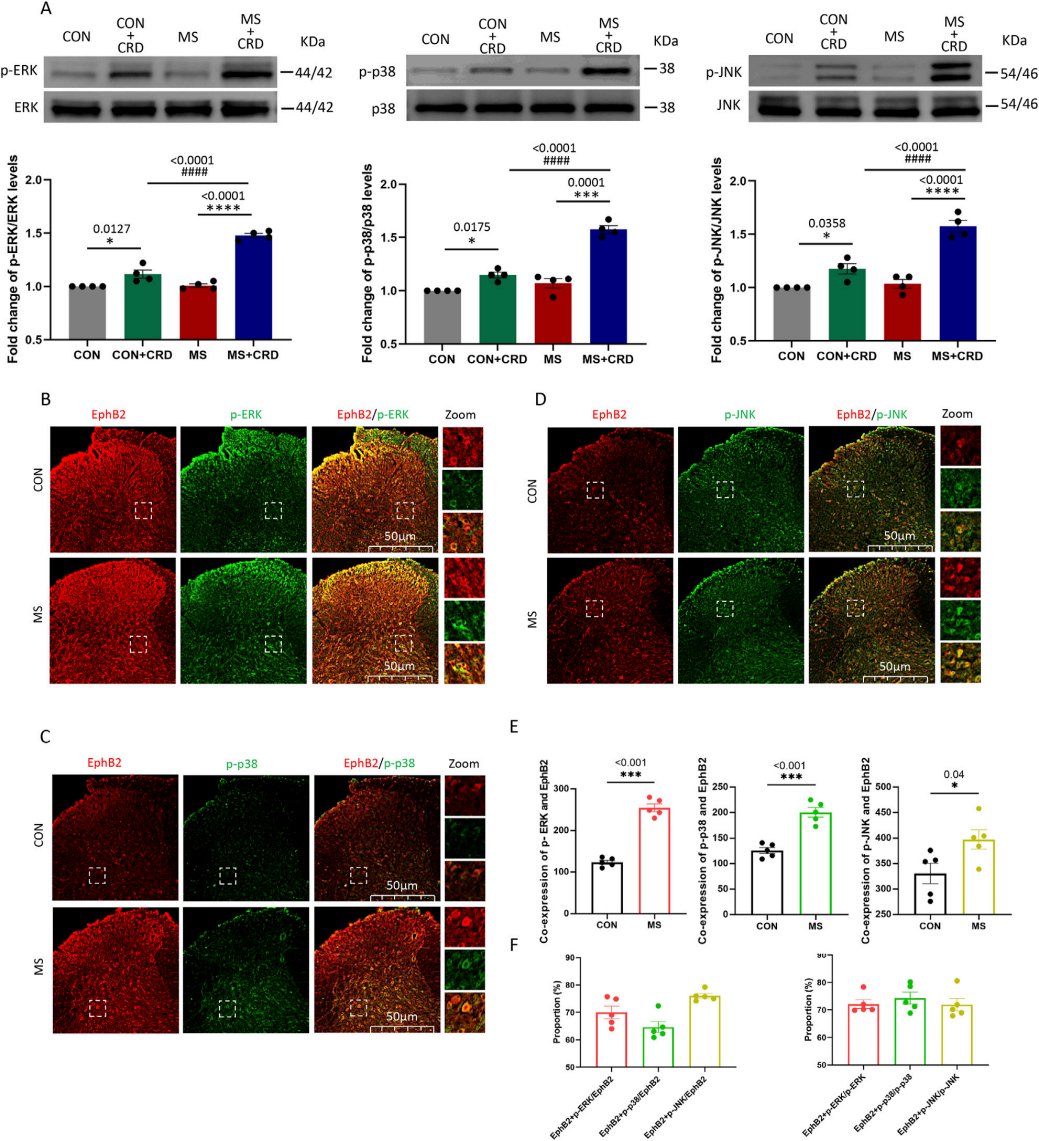
Visceral hyperalgesia in response to colorectal distension activates EphrinB2/EphB2 signaling and downstream MAP kinases. Mice were subjected to maternal separation (MS) early in development or not (CON), then later subjected to colorectal distension (CRD) or not. **(A)** Representative western blots of total lysate from spinal cord and quantification of the active, phosphorylated forms of the MAP kinases ERK, p38 and JNK. Levels of phosphorylated protein were normalized to those of the corresponding total protein, and the relative level of phosphorylated protein in the control group without CRD was defined as 1. Quantification is shown for four animals per condition. Based on one-way ANOVA and the Bonferroni multiple-comparisons test: *P < 0.05, ****p < 0.0001 between CON and CON + CRD or between MS and MS + CRD; ^####^ *P* < 0.0001 between CON + CRD and MS + CRD. **(B–D)** Immunostaining of thin sections of spinal cord against EphB2 (red) and the phosphorylated forms of p38, ERK, or JNK (green). Scale bar, 50 µm. The boxed regions in the large images are shown at higher magnification on the *far right* (“Zoom”). **(E)** Co-expression of spinal EphB2 and MAPKs. Based on one-way ANOVA and the Bonferroni multiple-comparisons test: *P < 0.05, ***P < 0.001 between CON and MS. **(F)** Proportion of spinal cord cells expressing each activated MAP kinase that also expressed EphB2 (*left plot*) or proportion of spinal cord cells expressing EphB2 that also expressed each of the phosphorylated MAP kinases (*right plot*).

In the published article, there was an error in Figures 4E, F as published. In Figures 4E, F “Actin” was incorrectly written as “GAPDH”. The corrected Figure 4 and its caption appear below.

**FIGURE 4:**
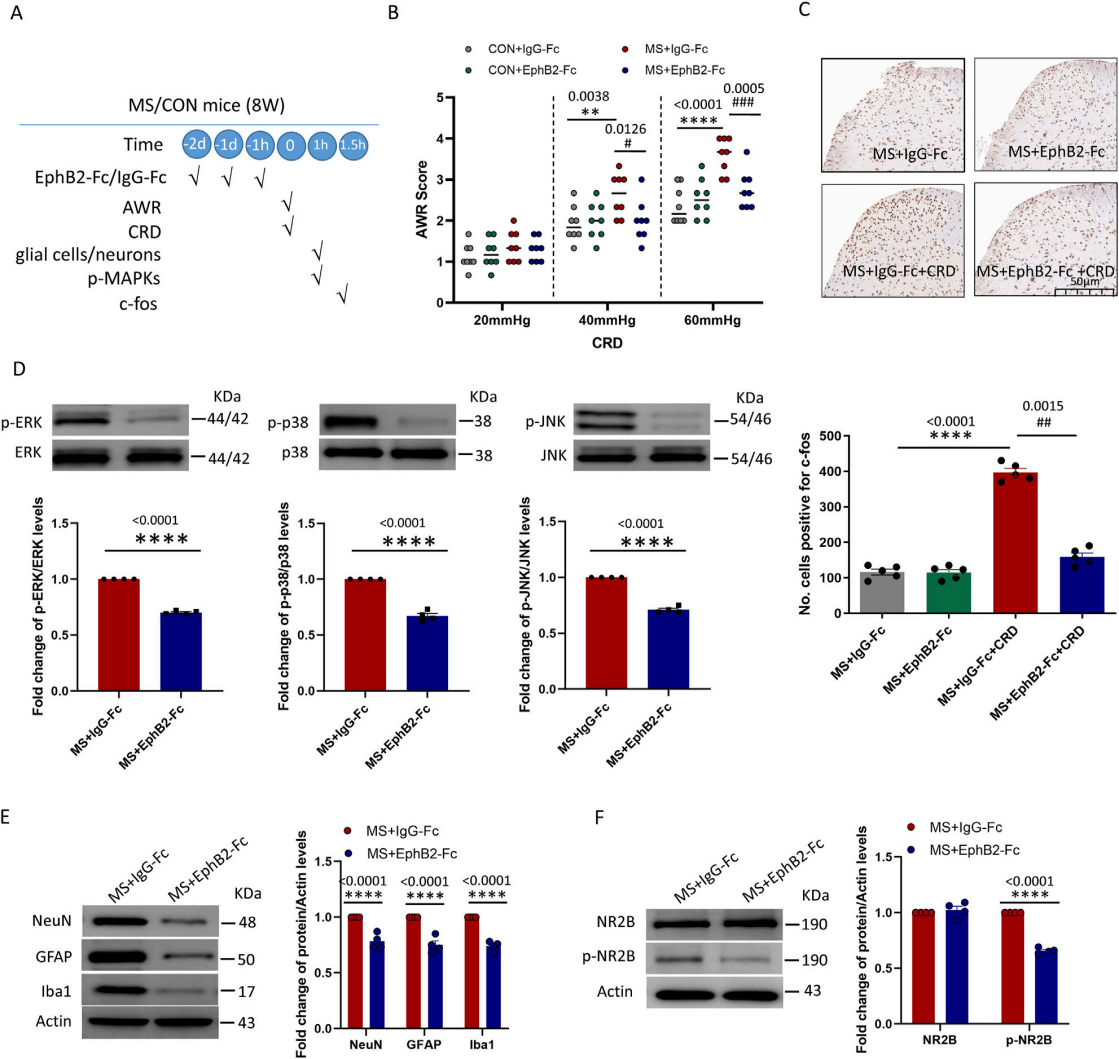
Inhibition of EphrinB2/EphB2 signaling mitigates the effects of maternal separation. **(A)** Schematic illustration of the experimental protocol. Mice were subjected to maternal separation (MS) early in development or not (CON), intrathecally injected with a chimera of EphB2 and Fc (EphB2-Fc) that inhibits EphB2 signaling or a negative-control chimera (IgG-Fc), then subjected to colorectal distension (CRD), during which the abdominal withdrawal reflex (AWR) was measured. Subsequently, the spinal cord was analyzed by Western blotting and immunohistochemistry to observe expression of key proteins. **(B)** AWR score during CRD. Quantification is shown for eight animals per condition. Based on two-way repeated-measure ANOVA and the Bonferroni multiple-comparisons test: **P < 0.01, ****P < 0.0001 between CON + IgG-Fc and MS + IgG-Fc; #*P* < 0.05, ###*P* < 0.001 between MS + IgG-Fc and MS + EphB2-Fc. **(C)** Representative micrographs of spinal cord tissue after immunostaining against c-fos and quantification of the number of cells expressing c-fos. We count the c-fos + neurons in lamine I-V of spinal cord of view at magnification ×10 and average the results. Results are shown for five animals per condition. Scale bar, 50 µm. Based on one-way ANOVA and the Bonferroni multiple-comparisons test: ****P < 0.0001 between MS + IgG-Fc and MS + IgG-Fc + CRD; ##*P* < 0.01 between MS + IgG-Fc + CRD and MS + EphB2-Fc + CRD. **(D–F)** Representative western blots of total lysate from spinal cord and quantification of **(D)** the active, phosphorylated forms of the MAP kinases ERK, p38 and JNK; **(E)** the cell type markers NeuN, GFAP and Iba1; or **(F)** NR2B and NR2B phosphorylated on Tyr1472. Levels of phosphorylated protein were normalized to those of the corresponding total protein, while levels of markers, NR2B or phospho-NR2B were normalized to those of Actin; the resulting relative protein levels in the MS+IgG-Fc group were defined as 1. Quantification is shown for four animals per condition. Based on unpaired t-test, ****P < 0.0001 compared with MS + IgG-Fc group.

In the published article, there was an error in Figures 5D, E as published. In Figures 5D, E “Actin” was incorrectly written as “GAPDH”. The corrected Figure 5 and its caption appear below.

**FIGURE 5:**
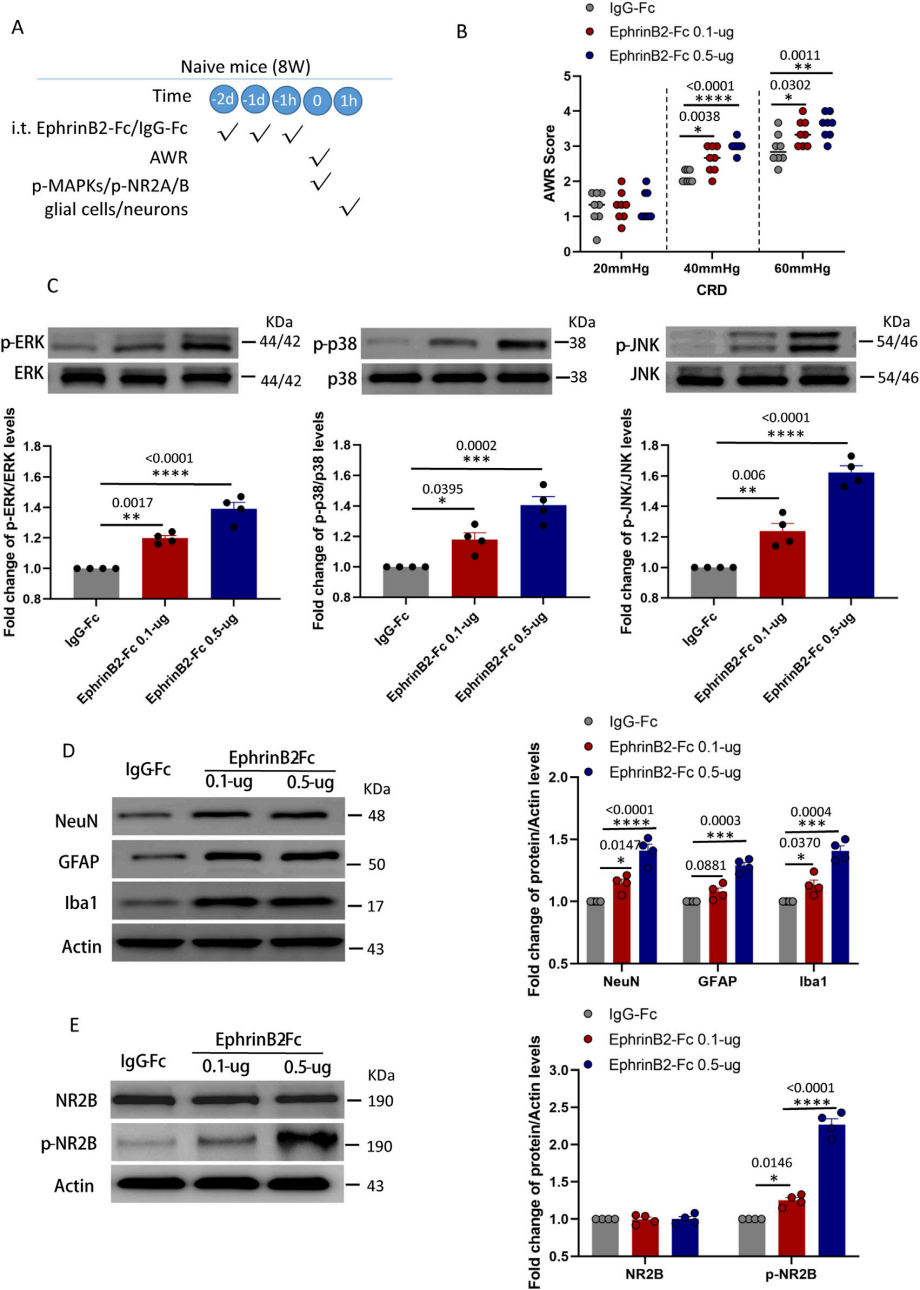
Activation of EphrinB2/EphB2 signaling reproduces the effects of maternal separation in naïve mice. **(A)** Schematic illustration of the experimental protocol. Mice that had not experienced maternal separation early in development were intrathecally injected with a chimera of EphrinB2 and Fc (EphB2-Fc) that activates EphB2 signaling or a negative-control chimera (IgG-Fc), then subjected to colorectal distension (CRD), during which the abdominal withdrawal reflex (AWR) was measured. Subsequently, the spinal cord was analyzed by Western blotting and immunohistochemistry to observe expression of key proteins. **(B)** AWR score during CRD. Quantification is shown for eight animals per condition. Based on two-way repeated-measure ANOVA and the Bonferroni multiple-comparisons test: *P < 0.05, **P < 0.01, ****P < 0.0001 vs IgG-Fc group. **(C)** Representative western blots of total lysate from spinal cord and quantification of the active, phosphorylated forms of the MAP kinases ERK, p38 and JNK. Levels of phosphorylated protein were normalized to those of the corresponding total protein, and the relative level of phosphorylated protein in the IgG-Fc group was defined as 1. Quantification is shown for four animals per condition. Based on one-way ANOVA and Bonferroni multiple comparisons test, *P < 0.05, **P < 0.01, ***P < 0.001, ****P < 0.0001 vs. IgG-Fc group, n = 4 mice in each group. **(D, E)** Representative western blots of total lysate from spinal cord and quantification of **(D)** the cell type markers NeuN, GFAP and Iba1; or **(E)** NR2B and NR2B phosphorylated on Tyr1472. Protein levels were normalized to those of Actin, and the relative protein level in the IgG-Fc group was defined as 1. Quantification is shown for four animals per condition. Based on one-way ANOVA and Bonferroni multiple comparisons test, *P < 0.05, ***P < 0.001, ****P < 0.0001 vs. IgG-Fc group.

The authors apologize for this error and state that this does not change the scientific conclusions of the article in any way. The original article has been updated.

