# Supplementary material for: Activation of EphrinB2/EphB2 signaling in the spine cord alters glia-neuron interactions in mice with visceral hyperalgesia following maternal separation
Source: Front Pharmacol. 2024 Sep 3;15:1463339. doi: 10.3389/fphar.2024.1463339 (PMC11405339; doi:10.3389/fphar.2024.1463339)
Supplement: Supplementary file 1 [file Image1.pdf]

Supplementary Figure 1D.

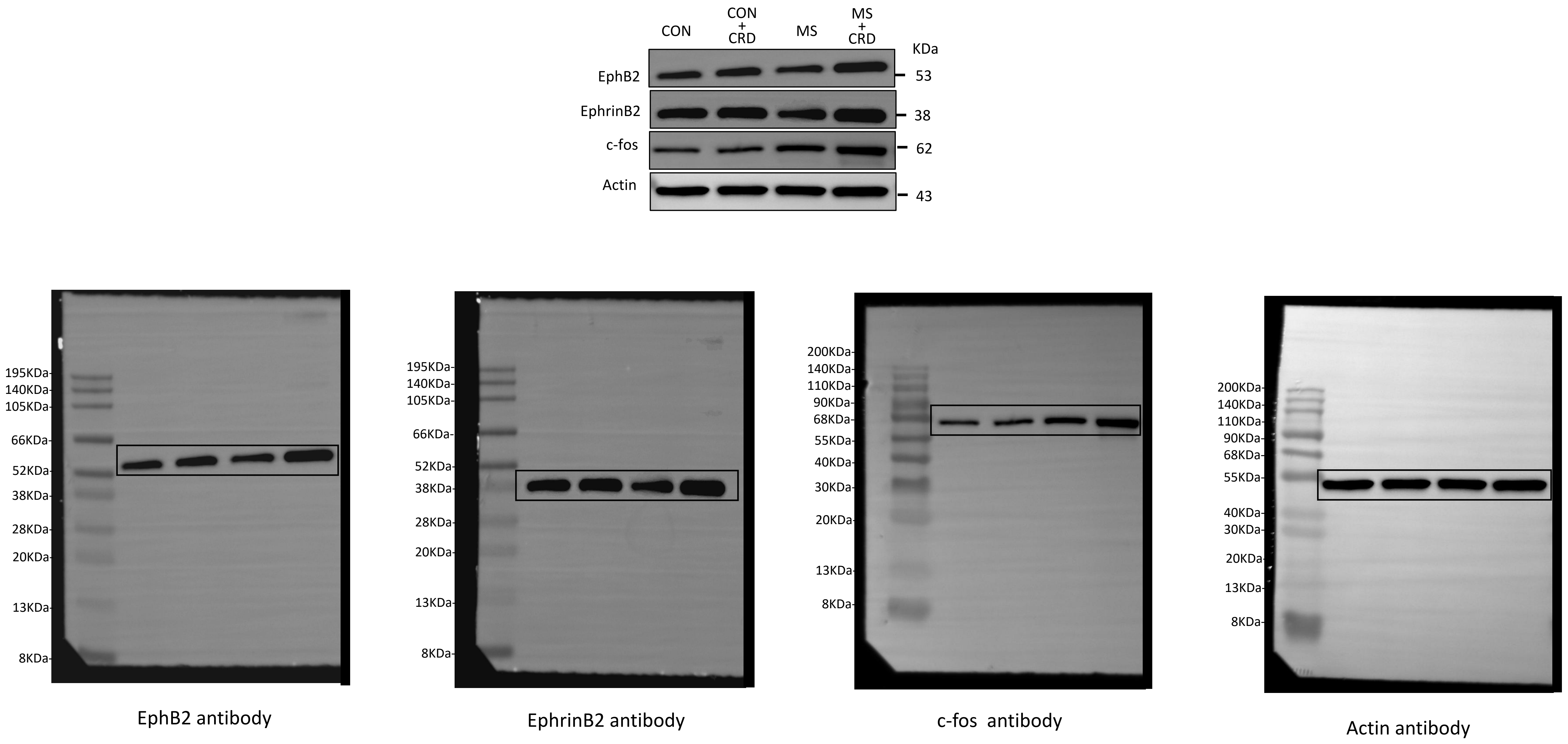

Supplementary Figure 1D. A full scan of the entire original gel(s).

Supplementary Figure 2A.

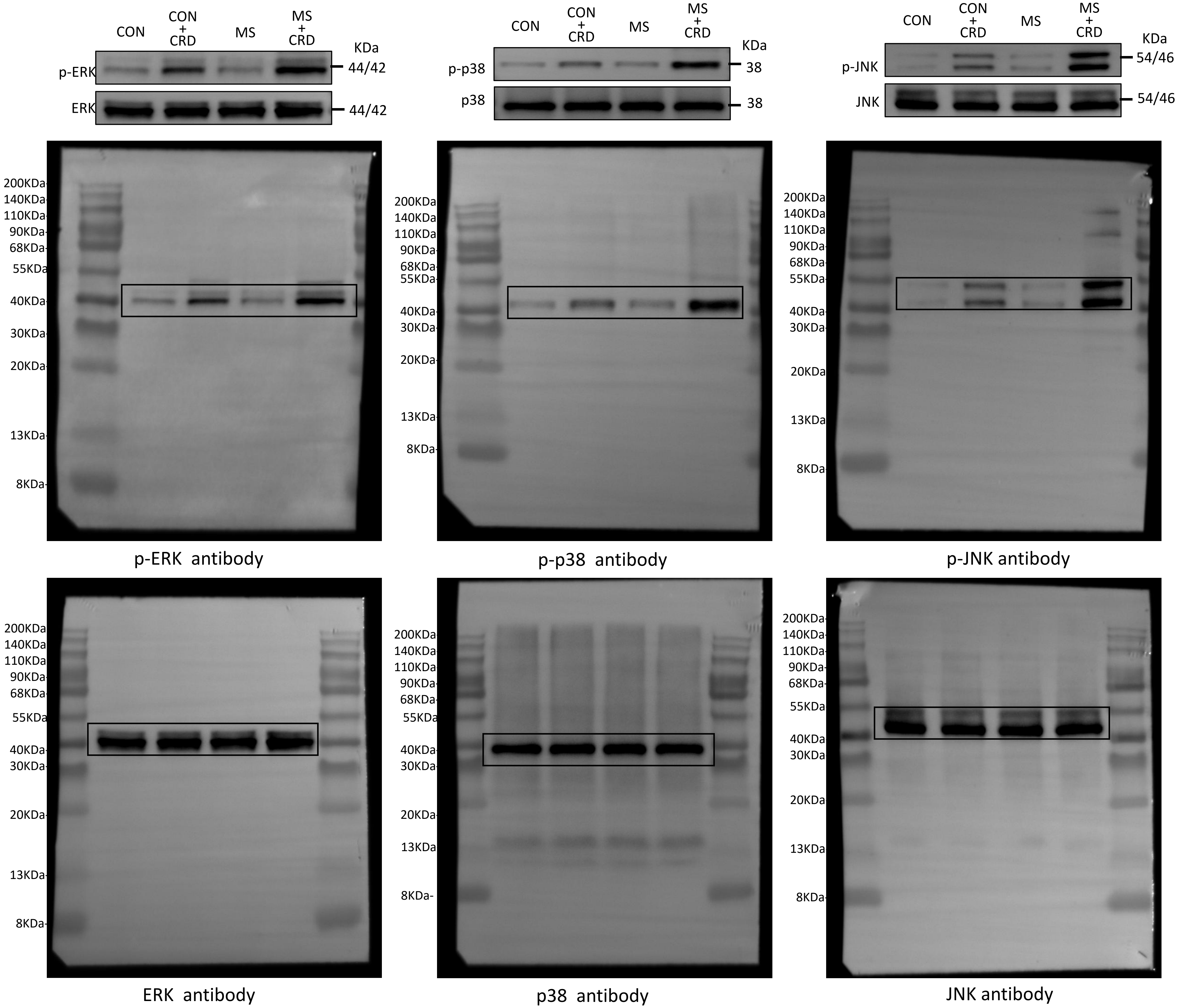

Supplementary Figure 2A. A full scan of the entire original gel(s).

Supplementary Figure 4D.

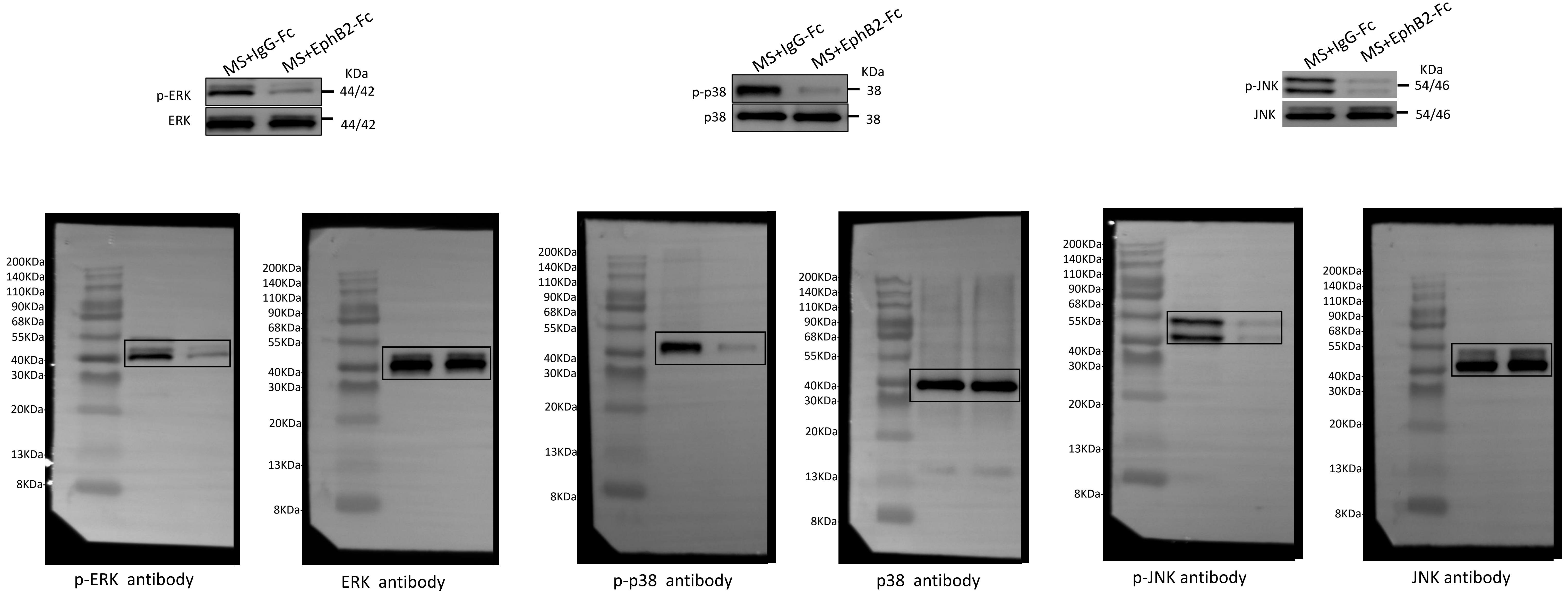

Supplementary Figure 4D. A full scan of the entire original gel(s).

Supplementary Figure 4E.

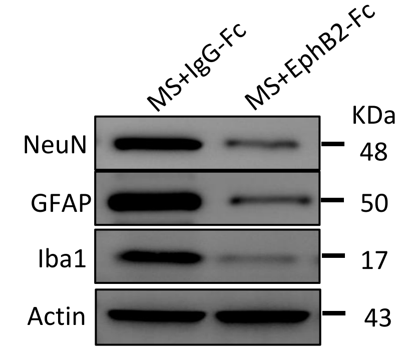

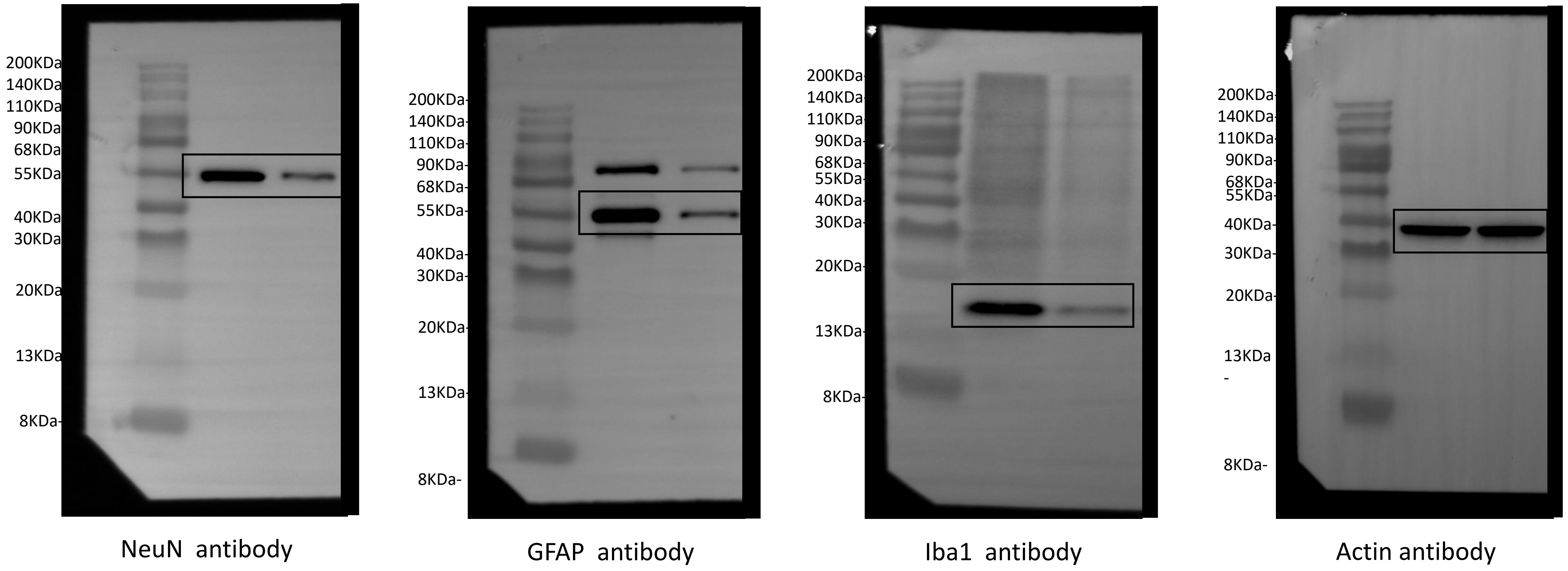

Supplementary Figure 4E. A full scan of the entire original gel(s).

Supplementary Figure 4F.

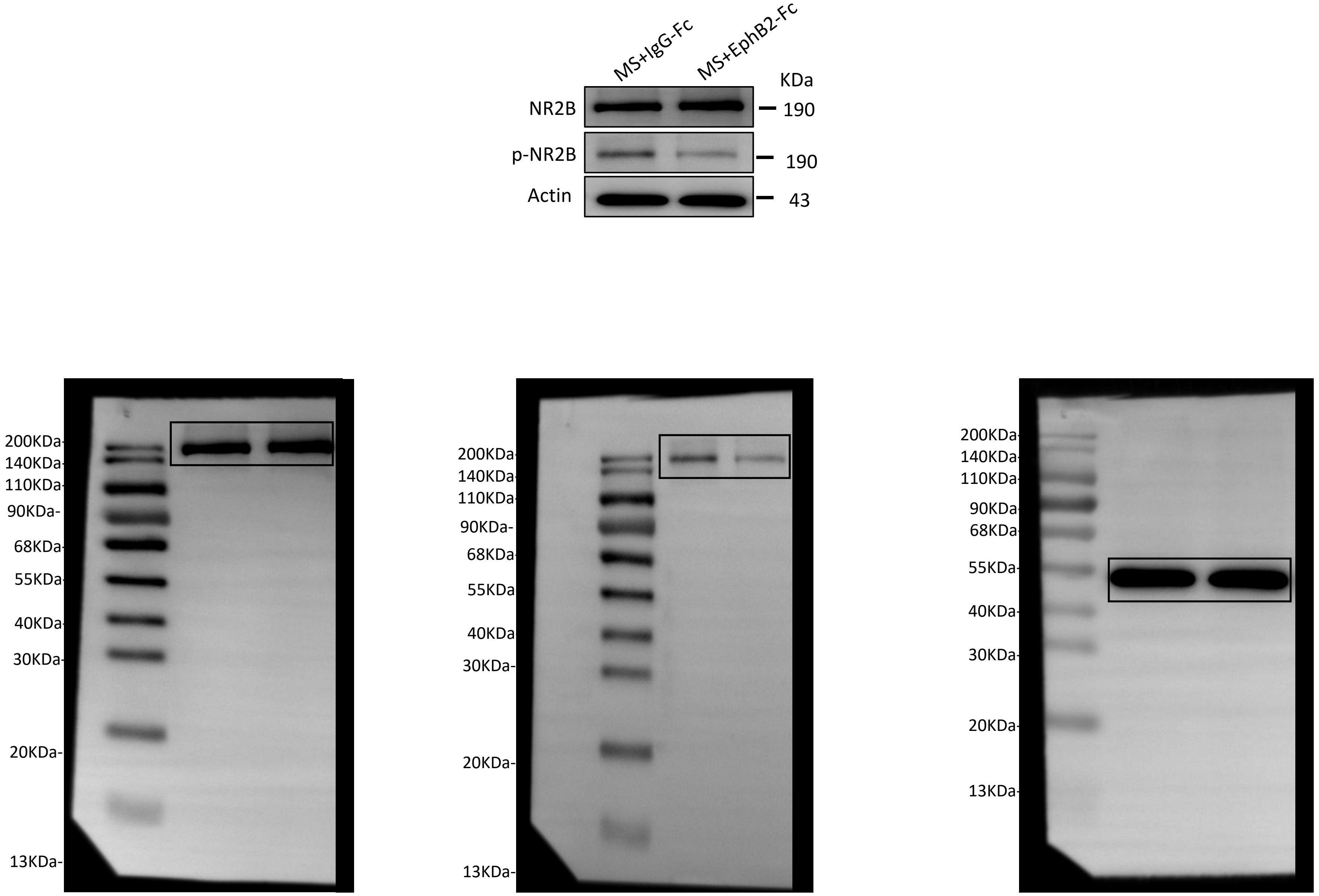

NR2B antibody   p-NR2B antibody   Actin antibody

Supplementary Figure 4F. A full scan of the entire original gel(s).

Supplementary Figure 5C.

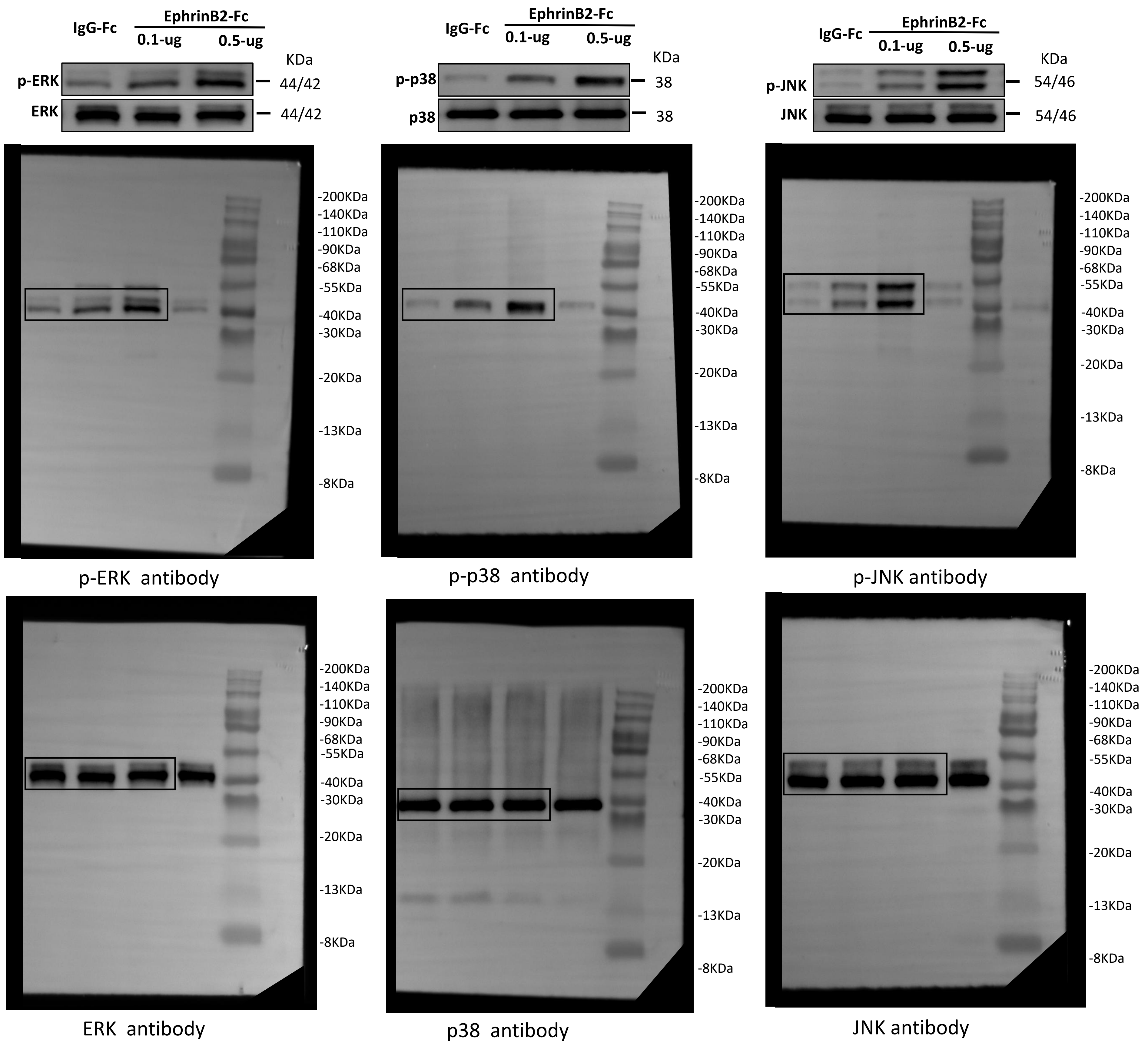

Supplementary Figure 5C. A full scan of the entire original gel(s).

Supplementary Figure 5D.

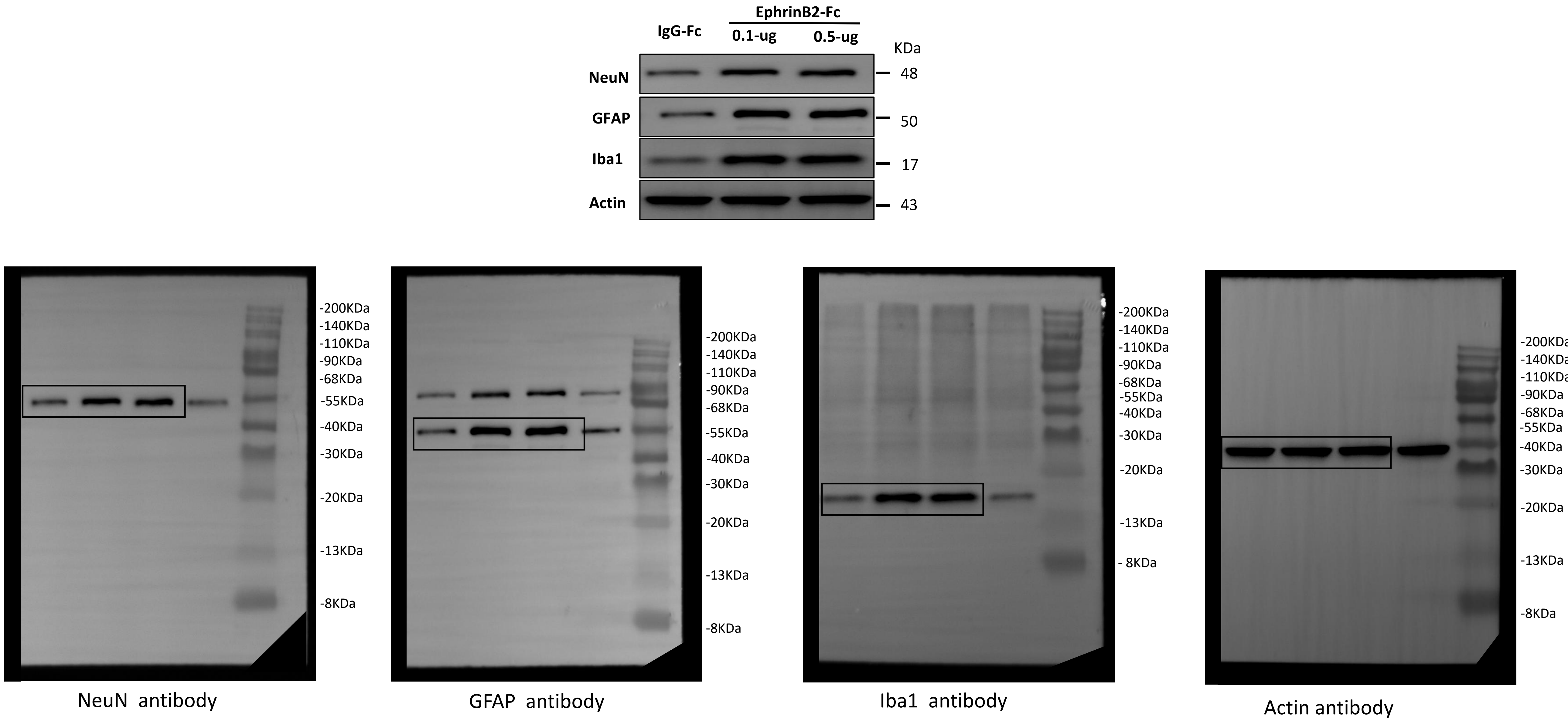

Supplementary Figure 5D. A full scan of the entire original gel(s).

Supplementary Figure 5E.

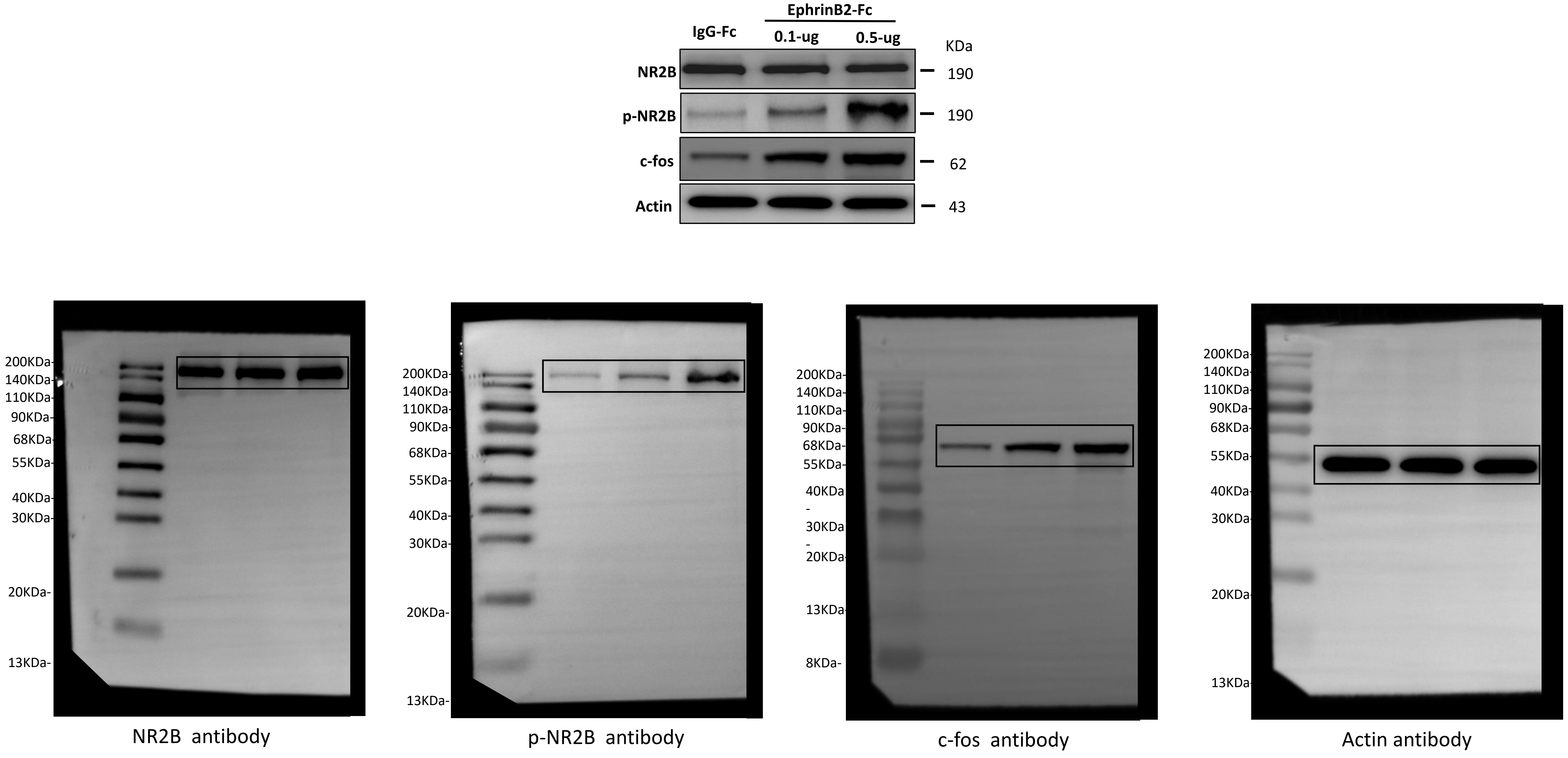

Supplementary Figure 5E. A full scan of the entire original gel(s).
